# Regulation of Phenolic Compound Production by Light Varying in Spectral Quality and Total Irradiance

**DOI:** 10.3390/ijms23126533

**Published:** 2022-06-10

**Authors:** Radomír Pech, Adriana Volná, Lena Hunt, Martin Bartas, Jiří Červeň, Petr Pečinka, Vladimír Špunda, Jakub Nezval

**Affiliations:** 1 Department of Physics, Faculty of Science, University of Ostrava, 710 00 Ostrava, Czech Republic; radomir.pech@osu.cz (R.P.); adriana.volna@osu.cz (A.V.); 2 Department of Experimental Plant Biology, Faculty of Science, Charles University, 128 00 Praha, Czech Republic; huntl@natur.cuni.cz; 3 Department of Biology and Ecology, Faculty of Science, University of Ostrava, 710 00 Ostrava, Czech Republic; martin.bartas@osu.cz (M.B.); jiri.cerven@osu.cz (J.Č.); petr.pecinka@osu.cz (P.P.); 4 Global Change Research Institute, Czech Academy of Sciences, 603 00 Brno, Czech Republic

**Keywords:** antioxidants, flavonoids, HPLC, miRNA, photoprotection, secondary metabolism, spectral quality of light, spring barley (*Hordeum vulgare*), transcriptomics, UV tolerance

## Abstract

Photosynthetically active radiation (PAR) is an important environmental cue inducing the production of many secondary metabolites involved in plant oxidative stress avoidance and tolerance. To examine the complex role of PAR irradiance and specific spectral components on the accumulation of phenolic compounds (PheCs), we acclimated spring barley (*Hordeum vulgare*) to different spectral qualities (white, blue, green, red) at three irradiances (100, 200, 400 µmol m^−2^ s^−1^). We confirmed that blue light irradiance is essential for the accumulation of PheCs in secondary barley leaves (in UV-lacking conditions), which underpins the importance of photoreceptor signals (especially cryptochrome). Increasing blue light irradiance most effectively induced the accumulation of B-dihydroxylated flavonoids, probably due to the significantly enhanced expression of the *F3*′*H* gene. These changes in PheC metabolism led to a steeper increase in antioxidant activity than epidermal UV-A shielding in leaf extracts containing PheCs. In addition, we examined the possible role of miRNAs in the complex regulation of gene expression related to PheC biosynthesis.

## 1. Introduction

Light is the source of energy which enables photosynthesis, but also serves as a source of environmental information influencing plant physiology, metabolism, and development. This information is derived from both spectral intensity and spectral quality, which vary depending on factors such as time of day/season [1,2], canopy position [3,4], and atmospheric conditions [5]. However, light can also be a stressor, as excessive photosynthetically active radiation (PAR) or ultraviolet (UV) irradiation leads to the overproduction of reactive oxygen species (ROS) and the subsequent state of oxidative stress. At appropriate levels, ROS also play an important role in plant physiology and development by regulating tip growth; modulating cell wall properties [6]; and acting as signaling molecules influencing stomatal activity [7], protective responses, plant stress acclimation, and programmed cell death [8,9,10]. At high levels, ROS interact with biomolecules, disrupting their structure and function via protein oxidation (particularly the proteins of Photosystem II [11,12]), changes in nucleic acid sequences [13], and lipid peroxidation, which can cause disturbances in the structural integrity of cellular and subcellular membranes [8,12,14]. Excessive ROS concentrations may thus cause many adverse effects, such as plant growth reduction, impaired development, or even the death of a whole plant.

Plants have developed many protective mechanisms to cope with adverse light conditions and excessive ROS production. While some mechanisms prevent ROS production (e.g., epidermal UV shielding, thermal dissipation of excess light energy, adjustment of leaf inner structure, chloroplasts movements, photosystem state transitions, etc.), others utilize compounds with antioxidative properties to scavenge already-produced ROS [12]. In non-stress conditions, the production of ROS is balanced by the scavenging activity of plant antioxidative systems. The plant antioxidant defense system, specifically the part responsible for eliminating ROS (ROS scavenging), consists of two functionally interconnected (coacting) components—enzymes with antioxidative function (superoxide dismutase—SOD, catalase—CAT, ascorbate peroxidase—APX, etc.) and low-molecular-weight antioxidants (LMWA), such as tocopherols, ascorbate, glutathione, carotenoids, and phenolic compounds (PheCs).

Antioxidative enzymes work in tightly linked systems. These enzymes are highly efficient (e.g., catalytic efficiency of SOD is approximately kcat/KM 7 × 10^9^ M^−1^ s^−1^), and their activity can be adjusted (for instance, the activity of SOD is driven by the concentration of H_2_O_2_ and superoxide anion radicals [15]). In addition to antioxidative enzymes, other LMWAs may be modulated by incident light, including PheCs and the enzymes involved in their biosynthesis. PheCs play a role in plant defense against a wide range of stressors [16]. This large group of secondary metabolites includes photoprotective compounds, such as flavonoids and anthocyanins (important subclasses of PheCs), which absorb strong UV and PAR when localized in the epidermis [17] and parenchyma [18].

**Figure 1:**
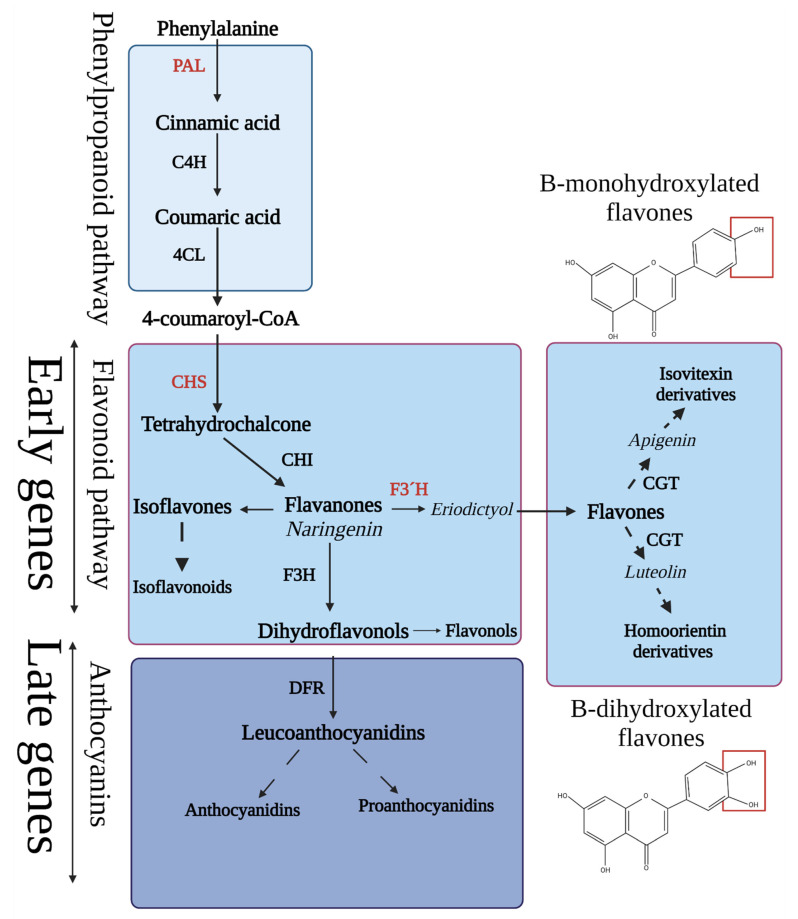
Simplified scheme of pathways responsible for phenolic compound biosynthesis with highlighted early and late gene groups encoding PheC-related enzymes. The expression of genes labeled in red was studied in this experiment. *PAL* (phenylalanine ammonia lyase; EC 4.3.1.24), *C4H* (cinnamate 4-hydroxylase; EC 1.14.13.11), *4CL* (4-coumarate CoA-ligase; EC 6.2.1.12), *CHS* (chalcone synthase; EC 2.3.1.74), *CHI* (chalcone isomerase; EC 5.5.1.6), *F3*′*H* (flavonoid 3′-hydroxylase; EC 1.14.14.82), *CGT* (c-glucosyltransferase; EC 2.4.1.360), *F3H* (flavanone 3-hydroxylase; EC 1.14.11.9), and *DFR* (dihydroflavonol 4-reductase; EC 1.1.1.219). Typical structures of B-monohydroxylated (apigenin) and B-dihydroxylated (luteolin) flavones (derivatives of which are present in soluble form in barley leaves) are shown and their structural difference is marked red.

Flavonoid synthesis requires precursors from several metabolic pathways: phenylalanine (from the shikimic acid pathway), which is further transformed to 4-coumaroyl-CoA within the phenylpropanoid pathway (Figure 1). The first reaction belonging to the flavonoid pathway itself occurs when 4-coumaroyl-CoA further reacts with malonate (from the malonic acid pathway). Flavonoids contain two aromatic benzene nuclei linked through a 3-carbon oxygen-containing heterocycle (C6-C3-C6 structure). Due to the presence of aromatic rings in their structure, flavonoids are effective UV attenuators. The accumulation of flavonoids in epidermal layers thus plays important role in plant UV tolerance (and avoidance of ROS production during UV stress). The efficiency of their antioxidant activity strongly depends on the configuration and the total number of hydroxyl groups, e.g., it is known that flavonoids containing a dihydroxylated B-ring, including homoorientin derivatives (such as luteolin and quercetin derivatives), may exhibit a higher antioxidant capacity than corresponding monohydroxylated PheCs [19]. In addition to antioxidant activity, PheCs suppress the formation of reactive species due to metal chelation, which prevents the formation of hydroxyl radicals produced by the Fenton reaction and may affect ROS signaling as well [20,21].

Biosynthesis of PheCs is tightly linked with PAR irradiance and spectral quality; thus, manipulation of light conditions can lead to changes in the content of these metabolites and consequently altered states of photoprotection [22]. Perception of light is ensured by photoreceptors, which can trigger a complex signaling cascade leading to acclimation and adaptive responses, induced at the level of gene expression (GE) via specific transcription factors (TFs) and their complexes [23]. UV-B and UV-A radiation stimulate PheC synthesis via the UV Resistance Locus 8 [24]. High intensities of PAR also induce PheC accumulation [25], especially blue light, which is perceived via cryptochromes (CRYs) [26,27]. Although phytochromes (PHY) are active primarily in the red region of light spectra [28], it was recently found that phytochromes in their active state (Pfr form) absorb photons also in the blue region [29,30]; thus, a role of PHY in blue-light-dependent regulation of PheCs synthesis cannot be excluded. Moreover, PHY may participate in the regulation of PheC biosynthesis through PIFs (PHYTOCHROME INTERACTING FACTORS), which can interact directly with CRY (PIF4 and PIF5) [31] or cooperate with TFs [32].

**Figure 2:**
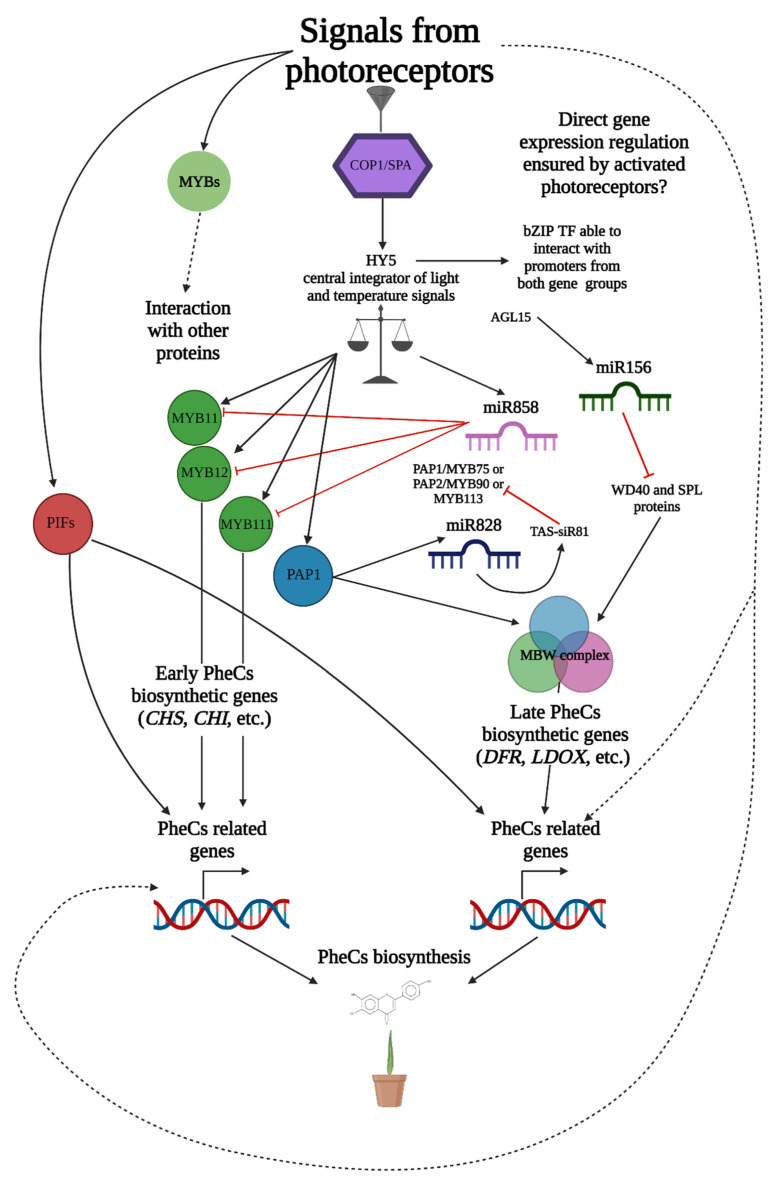
Simplified schematic representation of genes related to PheC biosynthesis and their regulation. Solid black arrows indicate known interactions, signaling, and regulatory mechanisms involved in PheC synthesis. Dashed black arrows mark other presumed regulatory pathways which are not yet confirmed. Red lines mark known downregulation or inhibitory interactions. TF (transcription factor), COP1/SPA complex (CONSTITUTIVELY PHOTOMORPHOGENIC 1, SUPPRESSOR OF PHYA-105); HY5 (ELONGATED HYPOCOTYL 5); PIFs (PHYTOCHROME INTERACTING FACTORS); miRNA (micro ribonucleic acid); MBW complex consisting of three proteins—MYB75 (PAP1), bHLH (TT8), and WD40 (TTG1); *CHS* (chalcone synthase; EC 2.3.1.74); *CHI* (chalcone isomerase, EC 5.5.1.6); *DFR* (dihydroflavonol 4-reductase; EC 1.1.1.219); and *LDOX* (leucocyanidin oxygenase; EC 1.14.11.19).

All the above-mentioned photoreceptors regulate PheC production mainly through the TF HY5 (ELONGATED HYPOCOTYL 5), which was proposed as an integrator of light and temperature signals [33,34,35] (Figure 2), regulating the expression of approximately 3000 genes in Arabidopsis [36]. A major repressor of HY5-related photomorphogenic responses is the COP1/SPA complex (CONSTITUTIVELY PHOTOMORPHOGENIC 1, SUPPRESSOR OF PHYA-105), responsible for HY5 degradation. Light-activated photoreceptors may directly interact with COP1/SPA and prevent HY5 ubiquitination due to changes in COP1/SPA functionality [37]. Once photoreceptors prevent HY5 degradation, the whole signaling pathway leads mostly to the increased transcription of PheC-related genes, which induces PheC production. The main targets of HY5 are sequences containing G-box, but also other motifs (T/G-box, E-box, GATA-box, ACE-box, Z-box, C-box, and even hybrid C/G or C/A boxes) [36].

The TF HY5 may consequently promote the expression of genes encoding enzymes involved in the early biosynthesis of flavonoids (chalcone synthase—CHS, chalcone isomerase—CHI, flavonoid 3′-hydroxylase—F3′H) and downstream anthocyanins (late biosynthetic genes including dihydrokaempferol 4-reductase, leucoanthocyanidin oxygenase, flavonol 3-O-glucosyltransferase) (Figure 1). Additionally, the expression of regulatory genes in the large MYB family of TFs, such as MYB12, MYB111, MYBD (MYB—like protein D), MYBL2 (MYB—like protein B), and MYB75 (also known as phosphatidic acid phosphatase 1—PAP1), can be stimulated by HY5 [38,39,40,41] (Figure 2). While TFs MYB11, MYB12, and MYB111 can promote the expression of flavonoid-related genes directly, expression of anthocyanins (downstream in the biosynthetic pathway) is driven via the MBW complex consisting of three proteins—MYB75 (PAP1), bHLH (TT8), and WD40 (TTG1) [33].

Another possible mechanism involved in the regulation of PheC synthesis includes the action of microribonucleic acids (miRNAs). This level of regulation is ensured by double-stranded miRNAs, which are later degraded to single-stranded miRNAs and incorporated into the RISC complex (RNA-induced silencing complex). Once the miRNA finds a complementary messenger ribonucleic acid (mRNA), the translation may be stopped, or the mRNA strand coding for functional protein is degraded [42]. PheC-related genes are regulated by multiple miRNAs (Figure 2) from families 156, 858, 828, and others [43,44] to ensure precise modulation of GE in response to environmental factors. However, studies focused on the connecting light-signaling and miRNA-driven regulation of PheC metabolism are still limited.

Many published works studying light-driven changes of PheC metabolism in plant tissues deal with the impact of UV radiation, the spectral composition of PAR, or the total irradiance of an individual PAR region, and their combinations. However, there is a lack of experiments examining the effects of the main spectral components of PAR (B, G, R) on PheC production (including regulation mechanisms) at various irradiance levels. This type of study is important, as photoreceptor responses may differ at various irradiances. Furthermore, PheC regulation may be affected indirectly by light-induced changes in the photosynthetic process (affecting the availability of assimilates for secondary metabolism, as well as overall ROS production). Finally, the spectral composition may differently affect plant growth and development (and in turn, sink/source ratios) at various irradiances. Therefore, we decided to perform comparative analyses of PheC profiles and GE to inspect how the individual spectral regions of PAR and total irradiance affect the production of these protective compounds. We also performed a basic analysis of parameters related to plant tolerance against light-induced stress, including the determination of UV-A epidermal shielding, the total antioxidant activity (AOX) of polar LMWAs, as well as a basic assessment of antioxidant enzyme activity (based on the expression of related genes).

## 2. Results

### 2.1. Accumulation of Soluble PheCs Induced by Light Differing in Total Irradiance and Spectral Composition

**Figure 3:**
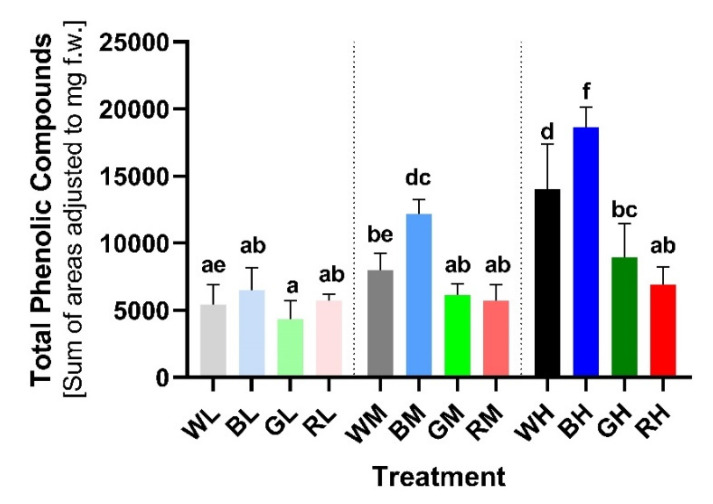
Total PheC content in secondary leaves of *Hordeum vulgare* L. cv. Bojos acclimated to light conditions varying in irradiance and spectral qualities. W (white), B (blue), R (red), G (green), L (low irradiance, 100 µmol m^−2^ s^−1^), M (medium irradiance, 200 µmol m^−2^ s^−1^), and H (high irradiance, 400 µmol m^−2^ s^−1^), *n* = 5–6 ± SD. The total content of soluble PheCs was evaluated based on HPLC-DAD data as a sum of peak areas (detected at 314 nm) and adjusted to the FW of each sample (for more details, see Section 4.4). Treatments marked above with same letters did not significantly differ based on Tukey’s *post-hoc* test.

To examine the effect of various light treatments on PheC accumulation and changes in their profile, HPLC (high performance liquid chromatography) analysis of leaf extracts was performed (Section 4.3 and Section 4.4). The total content of soluble PheCs (proxied by the sum of peak areas of individual compounds detected at 314 nm) varied significantly among tested light treatments. Plants exposed to low light intensity (LI; 100 μmol m^−2^ s^−1^) exhibited the lowest concentration of PheCs (Figure 3). Moreover, plants acclimated to various spectral qualities at LI conditions did not exhibit statistically significant differences in total PheC content (based on Tukey’s post-hoc test multiple comparisons—TPT). Although the BL plants contained a slightly higher concentration of PheCs compared to other spectral treatments at LI, the effect of spectral quality was negligible.

Plants exposed to medium light intensity (MI; 200 μmol m^−2^ s^−1^) exhibited different levels of PheC accumulation based on spectral quality. Comparing irradiance intensity (LI vs. MI), the only significant increase in PheC content was observed for the blue spectral treatment (BM vs. BL: +87.61%, TPT *p* < 0.0001). Plants grown under BM conditions exhibited the highest PheC content among plants cultivated at MI. Although plants acclimated to WM and GM treatments exhibited an increase compared to corresponding LI variants, it was not considered statistically significant (WM vs. WL: +46.26%, TPT ns; GM vs. GL: +41.34%, TPT ns). The red light MI did not cause any difference in PheC content (RM vs. RL: −0.82%, TPT ns).

Plants acclimated to high light intensity (HI; 400 μmol m^−2^ s^−1^) exhibited the highest total PheC content among all irradiance treatments (i.e., MI, LI); however, this difference was the most pronounced (and statistically significant) in conditions containing a higher proportion of blue spectral component (i.e., B, W). The highest accumulation of PheCs was observed in BH treatment (BH vs. BL: +187.66%, TPT *p* < 0.0001) and subsequently WH (WH vs. WL: +157.84%, TPT *p* < 0.0001). Surprisingly, a positive—yet less pronounced—effect of green light was also observed (GH vs. GL: +106.70%, TPT *p* = 0.0018). The effect of the red light was negligible regardless of the irradiance level (RH vs. RL: +20.23%, TPT ns). Based on the results of two-way ANOVA, we confirmed that both observed factors–irradiance and spectral quality—as well as their interaction had a statistically significant effect on the total soluble PheC accumulation (in all cases *p* < 0.0001, Supplementary Table S1).

### 2.2. Changes in the Profile of PheCs Caused by Different Light Conditions

**Figure 4:**
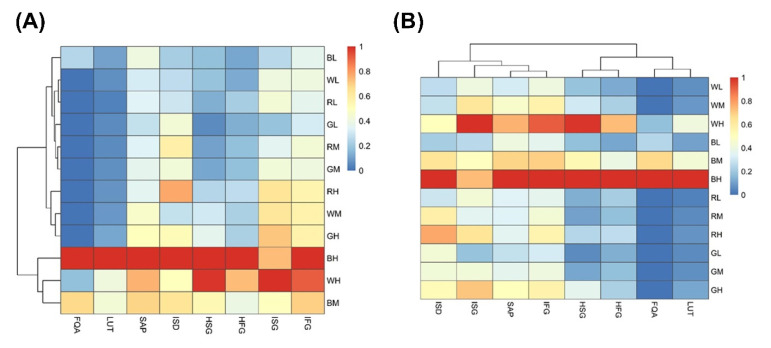
Heatmaps depicting similarities of PheC profiles among light treatments (**A**) as well as similarities in response of individual PheCs to the same light conditions (**B**). The relative contents of each PheC per treatment were averaged (*n* = 5–6) and subsequently normalized to the maximum value among all light treatments. Cluster analysis was performed on normalized PheC quantitative data (distance function: Euclidean distance; linkage function: Average linkage). Specifications of light treatments: W (white), B (blue), R (red), G (green), L (low irradiance 100 µmol m^−2^ s^−1^), M (medium irradiance 200 µmol m^−2^ s^−1^), and H (high irradiance 400 µmol m^−2^ s^−1^). Compounds of interest: FQA (feruloylquinic acid), LUT (lutonarin), SAP (saponarin), ISD (isoscoparin derivative), HSG (homoorientin-7-O-[6-sinp]-glc), HFG (homoorientin-7-O-[6-fer]-glc), ISG (isovitexin-7-O-[6-sinp]-glc), and IFG (isovitexin-7-O-[6-fer]-glc).

Figure 4 shows an overview of the changes in PheC profiles induced by varying light conditions in spring barley as heatmaps, including the results of a cluster analysis. The determined PheCs were tentatively identified (Supplementary Section S1: Identification of soluble phenolic compounds) as: FQA (feruloylquinic acid), LUT (lutonarin), SAP (saponarin), ISD (isoscoparin derivative), HSG (homoorientin-7-O-[6-sinp]-glc), HFG (homoorientin-7-O-[6-fer]-glc), ISG (isovitexin-7-O-[6-sinp]-glc), and IFG (isovitexin-7-O-[6-fer]-glc). The first heatmap (Figure 4A) depicts the similarities of whole profiles among light treatments, which are divided into two main clusters. The first cluster (C1) is formed by plants grown in the spectral conditions containing higher irradiances of blue light, such as BM, WH, and BH variants, which exhibited the highest relative concentration of PheCs in the data set (Figure 4A). These plants are characterized by the presence of FQA and a higher content of homoorientin derivatives, such as LUT, HSG, and HFG, compared to other treatments.

The second cluster (C2) is separated into two subclusters (SC). SC1 is formed by the LI and MI treatments (BL, WL, RL, GL, RM, and GM), in which low PheC content was typical regardless of spectral quality (Figure 4A). The samples belonging to BL treatment form an independent group within SC1 due to the slightly higher relative content of PheCs (mainly SAP and FQA). SC2, which consists of RH-, WM-, and GH-treated samples, exhibits low content of homoorientin derivatives (LUT, HSG, HFG) and FQA similarly to SC1, but (contrary to SC1) high content of SAP, ISG, and IFG.

In the second heatmap (Figure 4B), individual PheCs are divided into two main clusters based on similarities in their responses to light intensity and spectral quality. The C1 consists of ISD, SAP (in the range of 63.72 to 77.87% of the total PheC content, Supplementary Table S4), and other isovitexin derivatives (ISG, ISF). The content of these compounds was less affected by the spectral quality of light and rather depends on the irradiance. Thus, LI treatments contained generally low concentrations of isovitexin derivatives compared to plants exposed to MI and HI conditions. By contrast, C2 is formed by FQA, LUT, HSG, and HFG, which exhibited a strong dependence on the proportion of blue spectral component. This is especially true of LUT and FQA, which did not occur in leaves of plants acclimated to the light without a blue component or occurred only in trace amounts.

**Figure 5:**
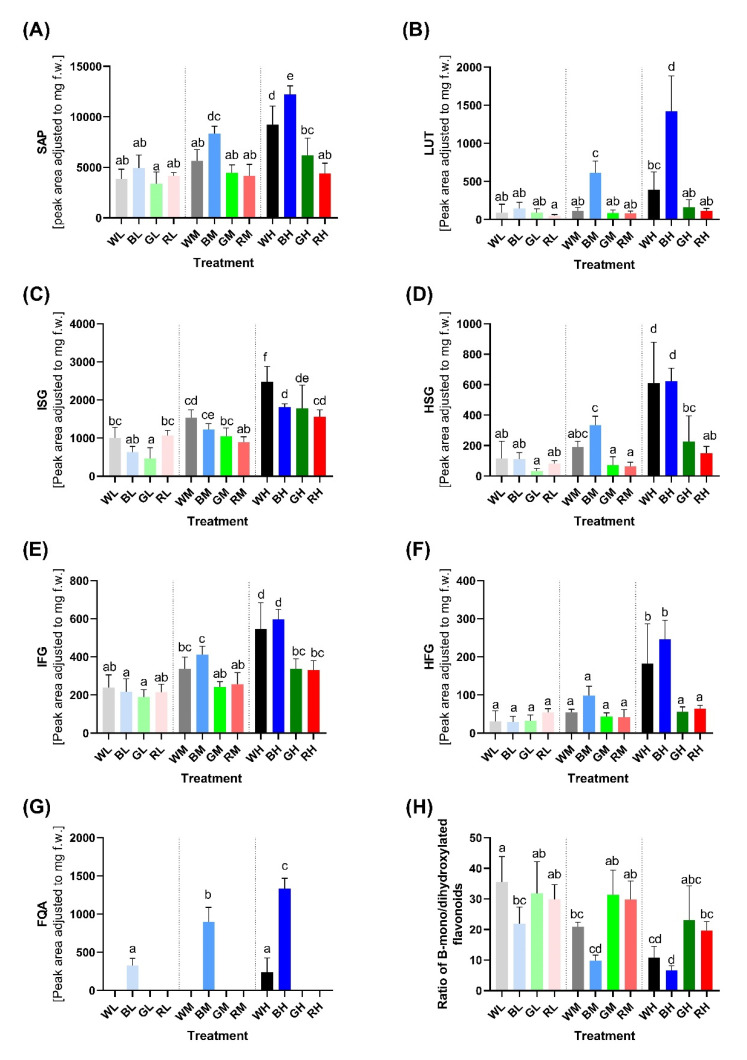
The relative content of individual PheCs in the secondary leaves of *Hordeum vulgare* L. cv. Bojos acclimated to the light conditions varying in irradiance and spectral qualities. Specifications of light treatment: W (white), B (blue), R (red), G (green), L (low irradiance, 100 µmol m^−2^ s^−1^), M (medium irradiance, 200 µmol m^−2^ s^−1^), and H (high irradiance, 400 µmol m^−2^ s^−1^); *n* = 5–6 ± SD; the relative content was determined for: (**A**)—SAP (saponarin), (**B**)—LUT (lutonarin), (**C**)—ISG (isovitexin-7-O-[6-sinp]-glc), (**D**)—HSG (homoorientin-7-O-[6-sinp]-glc), (**E**)—IFG (isovitexin-7-O-[6-fer]-glc), (**F**)—HFG (homoorientin-7-O-[6-fer]-glc), (**G**)—FQA (feruloylquinic acid), and (**H**)—Ratio of B-mono/dihydroxylated flavonoids. Treatments marked above with same letters did not significantly differ based on Tukey’s *post-hoc* test.

These results are illustrated in more detail in Figure 5, which contains a comparison of isovitexin derivatives (Figure 5A,C,E) with the corresponding B-dihydroxylated counterparts (Figure 5B,D,F). The relative content of isovitexin derivatives in plants was many times higher than homoorientin derivatives under all tested conditions. However, the strong positive effect of blue light on the accumulation of homoorientin derivatives led to a decrease in the isovitexin/homoorientin ratio in plants acclimated to HI (Figure 5H; WH, BH vs. WL, BL). A similar trend is visible also for plants acclimated to green and red light, although the decrease in the ratio of isovitexin/homoorientin derivatives with increasing irradiance is statistically insignificant. It is noteworthy that detectable accumulation of FQA was observed only in plants acclimated to blue light and under WH conditions.

### 2.3. Light Regulation of Epidermal UV-A Shielding

**Figure 6:**
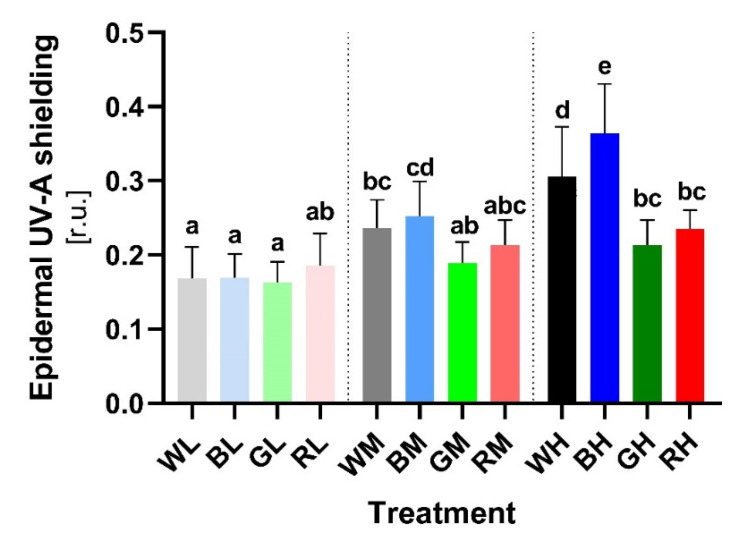
Epidermal UV-A shielding of *Hordeum vulgare* L. cv. Bojos secondary leaves acclimated to light conditions varying in irradiance and spectral qualities. Specifications of light treatments: W (white), B (blue), R (red), G (green), L (low irradiance, 100 µmol m^−2^ s^−1^), M (medium irradiance, 200 µmol m^−2^ s^−1^), and H (high irradiance, 400 µmol m^−2^ s^−1^); *n* = 10–12 ± SD. Treatments marked above with same letters did not significantly differ based on Tukey’s *post-hoc* test.

In vivo measurement of epidermal UV-A shielding determined by Dualex (Section 4.2) reflects the PheC content in the epidermal layer of secondary leaves (flavonoids/flavonols mainly). The lowest epidermal UV-A shielding was observed in plants acclimated to LI conditions (Figure 6). The shielding index among LI plants of various spectral qualities was comparable and statistically (also biologically) insignificant. Thus, the spectral quality at the low level of irradiance had a negligible effect on the UV-A transmittance of the epidermis.

On the contrary, the effect of spectral quality on the epidermal UV-A shielding was significant in plants acclimated to MI. Whereas BM and WM caused accumulation of UV-A shielding epidermal phenolics (BM vs. BL: +48.89%, TPT *p* < 0.0001; WM vs. WL: +40.01%, TPT *p* = 0.0027), no significant difference was observed under GM and RM conditions (GM vs. GL: +15.56%, TPT ns; RM vs. RL: +14.95%, TPT ns). Within MI, the blue-light-acclimated plants exhibited slightly higher epidermal UV-A shielding compared to other spectral treatments (mainly to GM).

Among all variants, the most effective induction of epidermal shielding occurred in plants cultivated in HI blue light (BH vs. BL: +114.93%, TPT *p* < 0.0001). The WH treatment exhibited a statistically significant positive effect on epidermal PheC induction (WH vs. WL: +81.32%, TPT *p* < 0.0001), although the effect was slightly weaker than observed for blue light. HI green light slightly increased shielding (GH vs. GL: +30.76%, TPT *p* = 0.004), followed by red light (RH vs. RL: +26.49%, TPT ns), the significant effect of which was not statistically confirmed. BH and WH plants revealed significantly higher epidermal UV-A shielding compared to GH- and RH-acclimated ones—such a pronounced effect of spectral quality was not observed under MI or LI.

The efficiency of UV-A epidermal shielding of secondary leaves showed a linear correlation with total soluble PheC content (measured on the same leaf segments as used for HPLC analysis, Supplementary Figure S4), and thus showed similar trends as the response to light treatments. Two-way ANOVA confirmed that spectral quality (*p* < 0.0001), irradiance (*p* < 0.0001), and their interaction (*p* < 0.0001) all had a statistically significant effect on epidermal UV-A transmittance (Supplementary Table S1).

### 2.4. Antioxidative Activity of Soluble PheCs

**Figure 7:**
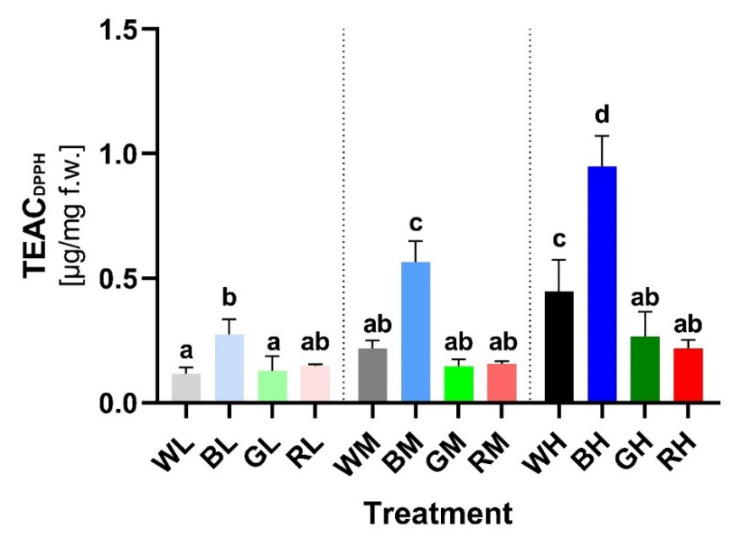
Antioxidant activity of soluble PheCs in secondary leaf extracts of *Hordeum vulgare* L. cv. Bojos acclimated to light conditions varying in irradiance and spectral qualities, expressed as a TEAC (Trolox-equivalent antioxidant capacity). Antioxidant activity was determined by colorimetric assay using 2,2-Diphenyl-1-picrylhydrazil stable radical (for details see Section 4.6). Specifications of light treatments: W (white), B (blue), R (red), G (green), L (low irradiance, 100 µmol m^−2^ s^−1^), M (medium irradiance, 200 µmol m^−2^ s^1^), and H (high irradiance, 400 µmol m^−2^ s^−1^); *n* = 5–6 ± SD. Treatments marked above with same letters did not significantly differ based on Tukey’s *post-hoc* test.

The lowest TEAC (Trolox-equivalent antioxidant capacity) measured spectrophotometrically using DPPH stable radical (Section 4.6) was observed in plants grown at LI. Although the average value was comparable within WL, RL, and GL groups, plants grown in BL exhibited a significantly higher TEAC, which suggests a positive effect of blue light on the antioxidative potential of plants, even at LI (Figure 7). This result differs from the effect of irradiance and spectral quality on total PheC content and epidermal UV-A shielding (Figure 3 and Figure 6), where statistical differences between LI treatments were not confirmed.

MI (200 μmol m^−2^ s^−1^) led to a significant increase in TEAC in BM-treated plants (BM vs. BL: +106.38%, TPT *p* < 0.0001) and contained a greater amount of PheCs compared to other MI variants (Figure 3). Negligible TEAC increases were observed in plants exposed to WL (WM vs. WL: +30.13%, TPT ns), followed by GM (GM vs. GL: +14.51%, TPT ns) and RM (RM vs. RL: +3.65%, TPT ns) conditions.

The highest TEAC was observed in plants acclimated to HI, but the TEAC values varied considerably compared to LI plants only in WH (WH vs. WL: +165.10%, TPT *p* < 0.0001) and especially BH (BH vs. BL: +246.37%, TPT *p* < 0.0001) treatments. These results suggest an important role of blue light in inducing the biosynthesis of PheCs with effective antioxidant properties. Exposure of plants to GH (GH vs. GL: +107.97%, TPT ns) and RH (RH vs. RL: +46.39%, TPT ns) did not lead to statistically significant differences compared to LI.

The TEAC values exhibited a linear dependence on PheC content (R = 0.9027, y = 5.401 × 10^−5^x − 0.1467, Supplementary Figure S3) and thus similar trends in response to acclimation light treatments. However, increasing irradiance of blue light enhanced the antioxidant activity of soluble PheCs more than the efficiency of epidermal UV-A shielding. The importance of spectral quality and irradiance on AOX of PheCs was confirmed by two-way ANOVA (*p* < 0.0001), including the interaction of these two factors (*p* < 0.0001) (Supplementary Table S1).

### 2.5. Expression Analysis of Genes Related to PheCs Biosynthesis, AOX Enzymes, and Senescence Markers

**Figure 8:**
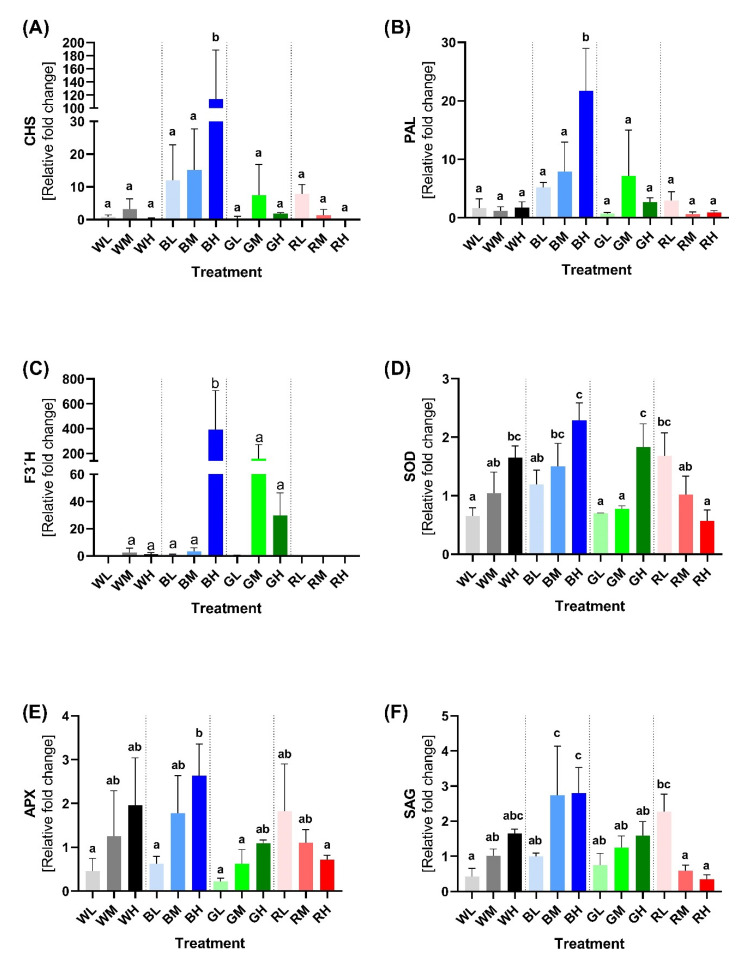
Results of RT-qPCR analysis of selected genes involved in the production of PheCs, antioxidant enzymes, and senescence in *Hordeum vulgare* L. cv. Bojos acclimated to light conditions varying in irradiance and spectral qualities. Specifications of light treatments: W (white), B (blue), R (red), G (green), L (low irradiance, 100 µmol m^−2^ s^−1^), M (medium irradiance, 200 µmol m^−2^ s^1^), and H (high irradiance, 400 µmol m^−2^ s^−1^); (**A**)—*CHS* (chalcone synthase, EC 2.3.1.74), (**B**)—*PAL* (phenylalanine ammonium lyase; EC 4.3.1.24), (**C**)—*F3*′*H* (flavonoid 3′hydroxylase; EC 1.14.14.82), (**D**)—*SOD* (superoxide dismutase; EC 1.15.1.1), (**E**)—*APX* (ascorbate peroxidase; EC 1.11.1.11), and (**F**)—*SAG* (senescence associated gene 12). Treatments marked above with same letters did not significantly differ based on Tukey’s *post-hoc* test.

We performed RT-qPCR analysis (Section 4.7 and Section 4.8) of three important genes involved in PheC biosynthesis: *PAL* (phenylalanine ammonia lyase), *CHS* (chalcone synthase), and *F3*′*H* (flavonoid-3′-hydroxylase) (see Figure 1 for their roles). We aimed to test whether the effects of light intensity and spectral composition on GE had the same trends as PheC accumulation. Indeed, our results showed a similar pattern to that described in Section 2.1. The highest PheC-associated GE was observed under blue light conditions, particularly under HI (Figure 8A,B). This suggests that total PheC accumulation occurs via both increased transcription after the seven-day acclimation and increased enzyme activity. This also indicates that blue light induces constitutively higher expression of PheC-related enzymes, even in the long term (Figure 8A,B). PAL catalyzes the synthesis of transcinnamic acid and CHS is responsible for the synthesis of naringenin chalcone. Upregulation of *PAL* and *CHS* was strongly dependent on spectral quality. Upregulation with increasing irradiance occurred only for plants acclimated under blue light (Figure 8). To determine how spectral quality and irradiation affect the expression of genes responsible for flavonoid hydroxylation, we also analyzed *F3*′*H*, which catalyzes the addition of hydroxyl groups to the flavonoid skeleton resulting in 3′-hydroxyflavonoid. The highest *F3*′*H* expression was observed in BH conditions, but also in GM and GH conditions. In all other conditions, *F3*′*H* expression was considerably weaker, but nonzero. ANOVA confirmed the statistically significant effect of spectral quality, irradiance, and their interaction for all analyzed genes (Supplementary Table S2).

To assess how spectral quality and irradiance of light influence the function of the key antioxidant enzymes, we determined gene expression of the SOD gene (encoding the superoxide dismutase), which converts superoxide radicals to hydrogen peroxide, and APX (encoding ascorbate peroxidase), which catalyzes the conversion of hydrogen peroxide to water. Increased GE of enzymes related to antioxidative defense occurs under blue light conditions as well (Figure 8). *SOD* expression was highly affected by spectral quality (*p* = 0.0003), irradiance (*p* = 0.0001), and their interaction (*p* < 0.0001) (Supplementary Table S2). Intriguingly, the expression of both AOX-related genes exhibited a positive correlation with increasing irradiance of white, blue, and green light. However, under red spectral treatment, the response to irradiance was the opposite—increasing irradiance of red light led to decreased *SOD* expression (Figure 8D). APX displayed a similar expression pattern—its expression was affected by spectral quality (*p* = 0.0216), irradiance (*p* = 0.0178), and the interaction of those two factors (*p* = 0.0147) (Supplementary Table S2).

It is known that light affects plant aging; therefore, we analyzed *SAG* (senescence associated gene 12), a senescence marker in Arabidopsis [45] and barley [46,47,48], to discover how spectral quality and irradiance influence leaf senescence. We showed that spectral quality (*p* = 0.0002), and the interaction of spectral quality and irradiance (*p* = 0.0001) affect the GE of *SAG*. On the other hand, the effect of total irradiance was not confirmed (*p* = 0.0897), which suggests that the spectral quality of incident light plays a major role in plant senescence. It is questionable whether this relationship can be observed at irradiances higher than 400 μmol m^−2^ s^−1^. Interestingly, the *SAG* expression pattern is very similar to genes encoding antioxidative enzymes (Figure 8F), which highlights the importance of this poorly understood gene in plants.

### 2.6. Transcriptomic Analysis of Genes Affecting the Production of PheCs

**Figure 9:**
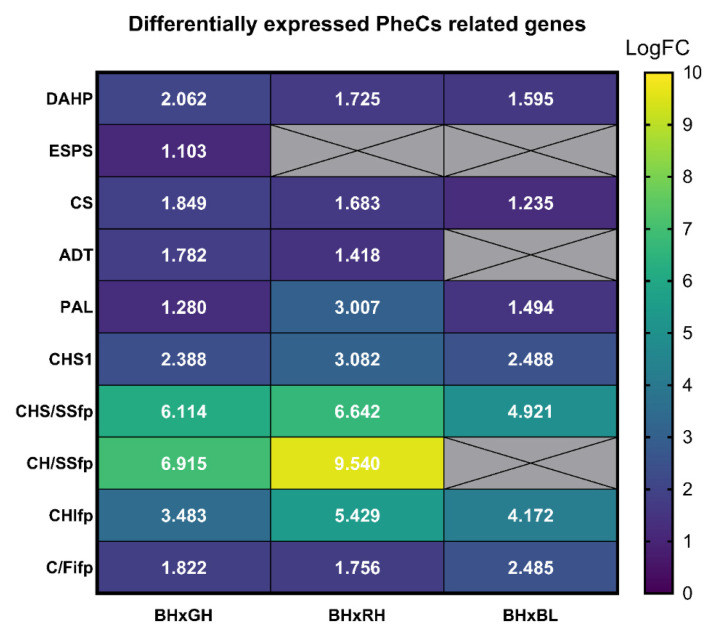
RNA sequencing analysis of PheC-related genes and genes of the shikimic pathway (which produces important substrates for PheC synthesis): Annotated genes are listed in rows from the top to the bottom in order of involvement in the biosynthetic pathways, whilst comparisons among light treatments are listed in columns (BH compared to GH, BH compared to RH, and BH compared to BL). Gray color denotes a missing value (in at least one of the datasets, the transcript was either not present or was excluded due to the parameters listed in Section 4.8). Displayed values correspond to logs of relative fold changes of gene expression (LogFC) between treatments and are also indicated by color according to the presented color scale. Specifications of light treatments: B (blue), R (red), G (green), L (low irradiance, 100 µmol m^−2^ s^−1^), and H (high irradiance, 400 µmol m^−2^ s^−1^). *DAHP* (3-deoxy-D-arabino-heptulosonic acid 7-phosphate synthase; MLOC_17364.2), *ESPS* (3-phosphoshikimate 1-carboxyvinyltransferase; MLOC_56626.1), *CS* (chorismate synthase; MLOC_66898.1), *ADT* (arogenate dehydratase; MLOC_65725.1), *PAL* (phenylalanine ammonia lyase; MLOC_79728.1), *CHS1* (chalcone synthase isoform 1; MLOC_74116.1), *CHS/SSfp* (chalcone/stilbene synthase family protein; MLOC_7936.1), *CH/SSfp* (chalcone/stilbene synthases family protein; MLOC_64305.2), *CHIfp* (chalcone-flavonone isomerase family protein; MLOC_5324.1), and *C/Fifp* (chalcone/flavonone family protein; MLOC_80571.3). MLOC IDs are unique identifiers in the STRING database (https://string-db.org/, accessed on 6 October 2021).

RNA-seq analysis revealed many differentially expressed genes among plants acclimated to BH, GH, and RH—due to the scope of this article, we focused on genes related to PheC biosynthesis (Figure 9). The comparison between BH and RH conditions revealed that BH showed upregulated *DAHP* (3-deoxy-D-arabino-heptulosonic acid 7-phosphate synthase), *CS* (chorismate synthase), *ADT* (arogenate dehydratase), *PAL* (phenylalanine ammonia lyase), *CHS1* (chalcone synthase isoform 1), *CHS/SSfp* (chalcone/stilbene synthase family protein), *CH/SSfp* (chalcone/stilbene synthases family protein), *CHIfp* (chalcone-flavonone isomerase family protein), and *C/Fifp* (chalcone/flavonone family protein) compared to RH. Similarly, a comparison between BH and GH light conditions revealed that *ADT*, *DAHP*, *CS*, *ESPS* (3-phosphoshikimate 1-carboxyvinyltransferase), *PAL*, *CHS1*, *CHS*, *CHS/SSfp*, *CHIfp*, and *C/Fifp* were significantly overexpressed in BH conditions. We also compared BH to BL growth conditions to find out whether irradiation affects PheC-related GE and found that in BH acclimated plants, *DAHP*, *PAL*, *CHS1*, *CH/SSfp*, *CHS/SSfp*, *CHIfp*, and *C/Fifp* were significantly overexpressed, but the enzymes involved in later steps of PheC biosynthesis were not.

Once we summarized our RNA-seq results, we could conclude that in BH conditions, genes related to the flavonoid pathway (so-called “early genes”; *CHS1*, *CHS/SSfp*, *CH/SSfp*, *CHIfp*, and *C/Fifp*) and genes from the phenylpropanoid pathway (specifically *PAL*) were significantly overexpressed, as well as some genes from the shikimic pathway (*DAHP*, *ESPS*, *CS*, and *ADT*). The greatest differences in GE between PheC biosynthesis genes were observed between BH and RH plants; the differences between BH and GH plants were less pronounced, and the least pronounced were the differences between BH and BL plants. All together, our analyses confirmed trends observed in the RT-qPCR analysis—the expression of PheC-related genes is proportional to blue light irradiance (BH vs. BL comparison) but also depends on the spectral quality (BH vs. RH, BH vs. GH)—the higher the wavelength of incident light, the lower the expression of these genes.

We also performed an analysis of differentially expressed small RNAs, including miRNAs. Our analysis revealed a whole scale of overexpressed small RNAs. The majority of them were snoR/snoZ/SNORD (small nucleolar RNAs responsible for chemical modifications of other RNAs), tRNAs (transfer RNAs involved in proteosynthesis), and rRNAs (responsible for ribosome constitution alongside specific proteins). However, those miRNAs are unable to induce post-transcriptional gene silencing (PTGS) and so were excluded from our detailed analysis. We discovered that miR1122 family members do not act uniformly in the context of different spectral quality and irradiance—some of them are overexpressed at BH conditions, while others are under-expressed (to avoid misinterpretation, their precise PLAZA IDs will be used further, Table 1). HVU0038G1818 and HVU0040G0316 were significantly overexpressed at BH conditions compared to GH and BL plants. A similar pattern was displayed by HVU0798G0114—this miRNA was overexpressed at BH conditions compared to GH and RH conditions.

In BH conditions, HVU0038G1160 and HVU0038G1161 from the miR1122 family, and also miR396 (HVU0042G1661), were underexpressed (Table 1) compared to GH and RH. Additionally, it is noteworthy that miR156 (HVU0042G2193) was significantly downregulated in BH compared to GH. MiR169_5 was also downregulated in BH conditions compared to RH and BL. On the other hand, two distinct isoforms (HVU0045G0592 in RH and HVU0037G2782 in BL) were preferred under those conditions. Together, our data suggest that miRNAs can also be differentially expressed at various light conditions to ensure an additional level of GE regulation and thus (indirectly) metabolic maintenance in suboptimal conditions, which is—to date (in the context of spectral quality and irradiation)— an undescribed phenomenon.

## 3. Discussion

### 3.1. Photosynthetically Active Radiation as an Important Factor Inducing PheC Biosynthesis and Plant Protective Mechanisms against Adverse Environmental Influences

Beyond powering photosynthesis, PAR plays a role in regulating plant defense to (photo)oxidative stress through the biosynthesis of PheCs. However, not all wavelengths contribute equally. Changes in spectral quality result in quantitative and qualitative changes to PheC profiles and, consequently, altered states of photoprotection [22]. There is increasing evidence that blue-light-induced accumulation of PheCs is a common plant response, as it was observed not only for *Hordeum vulgare* [49], but also in several other plant species: *Lactuca sativa* [50,51], *Chrysanthemum morifolium* [52], *Pisum sativum* [53], *Stevia rebaudiana* [54], *Eruca sativa* [55], and *Cucumis sativus* [56]. A study on Arabidopsis mutants with impaired CRY1 (blue-light-sensing) function showed significantly lower resistance against UV-B radiation due to the limited accumulation of UV-shielding compounds, but also lower catalase and peroxidase enzyme activity [57].

Importantly, the final plant response to photoreceptor-induced signals always depends on the other environmental stimuli as well as their mutual interaction (coaction) [58]. It is reasonable to investigate the interaction of light intensity and spectral quality since the variations of these two factors affect photoreceptor function itself, as well as their signaling pathways and, conclusively, PheC metabolism and its role in plant tolerance against oxidative stress.

### 3.2. PheC Production Is Effectively Enhanced by Blue Light but Not by Other Spectral Components of PAR during Acclimation of Spring Barley to Higher Irradiances

In our study, the acclimation of spring barley plants to the varying irradiance and spectral composition of PAR had a pronounced impact on soluble PheC metabolism. This involved changes in the total PheC content (Figure 3) as well as changes in the relative quantity of individual compounds (Figure 4 and Figure 5). PheCs accumulated in response to increasing PAR irradiance, but only if the acclimation light treatment involved a substantial blue spectral component. Increasing irradiance of blue light itself, i.e., in the absence of light belonging to the other spectral regions (UV-B, UV-A, G, R, FR) was sufficient to activate the PheC pathway and led to the most pronounced accumulation of PheCs among all treatments. However, results showed that interaction/co-action of blue-light-driven regulation of PheC metabolism and different irradiance levels might have important eco-physiological implications. For example, acclimation of spring barley to blue light of low irradiance 100 μmol m^−2^ s^−1^ did not enhance the quantity of PheCs compared to the other spectral qualities (Figure 3). Based on our results, we presume that the minimal total irradiance of blue light allowing effective accumulation of PheCs lies within 100–200 μmol m^−2^ s^−1^ in the case of spring barley (and the experimental conditions used). This may underpin the importance of UV radiation for initiating plant protective mechanisms against oxidative stress in forest understory or very dense canopies, where the blue light irradiance might not reach the necessary threshold level for PheC production. A minor yet non-negligible enhancement of total PheC accumulation was observed after acclimation of barley plants to HI green light (compared to GL; Figure 3). This observation was rather unexpected because blue-light-dependent PheC biosynthesis is driven to a high extent by CRYs induced signals [59]. It is known that green light can partially inactivate CRYs, presumably as a consequence of chromophore shifts to fully reduced FADH_2_ form [60]. Thus, green light is usually considered a signal to stop or slow down responses caused by activated CRY, including stem growth rate inhibition, anthocyanin accumulation, or chloroplast GE [61], which corresponds to results reporting a negligible effect of green light on PheC accumulation [62]. One possible explanation for the slightly stimulating effect of increasing green light irradiance on PheC biosynthesis in our conditions is the spectral overlap of green LED into the blue spectral region, which could activate CRYs (green LED spectral range 480–600 nm, Supplementary Figure S6). Although the primary absorption peak of cryptochromes in Arabidopsis corresponds approximately to 450 nm, absorption of FAD embedded in cryptochromes reaches up to 550 nm [63]. The green light may also affect cryptochromes localized deeper in the plant tissue due to its higher transmission compared to blue light [64,65]. Thus, the overlap of green LEDs to the blue spectral region could cause a change in the equilibrium of activated and inactivated CRYs, which in turn could slightly enhance total PheC content. Several studies have demonstrated the positive effect of green light on ascorbic acid, anthocyanin, and total phenolic content in lettuce [65,66,67]. However, Zhang et al. (2021) [68] concluded that green light reduces stem elongation when partially replacing blue light independent of CRY signaling. These results indicate that the green light responses may be induced also via CRY-independent pathway(s), which can contribute also to the observed enhancement of PheC accumulation in barley leaves.

### 3.3. Changes of PheC Profiles under Various Light Treatments—Blue Light as the Main Component of PAR Affecting the Ratio of B-Mono and Dihydroxylated Flavonoids

Aside from the total content of soluble PheCs, PAR irradiance and its spectral quality also had a significant impact on the PheC profile (i.e., relative content of PheCs within the sample). The soluble PheCs contained in barley secondary leaves could be divided into two distinctive groups according to their response to irradiance and spectral quality (Figure 4B). Isovitexin derivatives (1st group) tended to increase with HI (and availability of assimilates), even if it did not contain a blue spectral component. Conversely, the accumulation of homoorientin derivatives and FQA (2nd group) in response to HI was much more reliant on the presence of blue light, and thus rather low amounts of these compounds were observed in RH and GH treatments (Figure 4A and Figure 5). The most abundant soluble PheC detected in barley leaf extract was saponarin (in accordance with Kaspar (2010) [17]). Herein, saponarin represents from 63.72 to 77.87% of the total soluble PheC content in barley leaves acclimated to different irradiance and PAR spectra (one of the first studies, Seikel and Geissman (1957) [69] estimated approximately 72%), and hence, isovitexin derivatives exceeded the content of homoorientin derivatives in all tested plants. Isovitexin derivatives (SAP, ISG, IFG) are also exclusively responsible for the above-discussed increase in PheCs in GH compared to GL condition (compare Figure 5A,C,E and Figure 5B,D,F). However, the contribution of isovitexin derivatives to the total content of soluble PheCs decreased in favor of homoorientin derivatives in response to higher irradiance of blue and white light, while the changes in the ratio of isovitexin and homoorientin derivatives were statistically insignificant in barley plants acclimated to increasing irradiance of green and red light (Figure 5H). A previous study on barley showed that UV and PAR treatments had a minor effect on saponarin, whereas lutonarin was markedly enhanced by high PAR and UV irradiances, which led to comparable or even slightly higher content of lutonarin compared to saponarin in young leaves of *Hordeum vulgare* [70]. Thus, it seems that the content of isovitexin and homoorientin derivatives may gradually level out under suitable light conditions.

Importantly, under LI, the content of observed PheCs was not significantly affected by spectral treatments. Whether a certain threshold irradiance of blue light must be reached to effectively activate relevant photoreceptor (CRYs) signaling, or whether effective PheC accumulation (and metabolic profile change) was limited by insufficient availability of assimilates (e.g., saccharose signaling)—or other factors influencing plant metabolism under LI—remains unknown. In general, the accumulation pattern of glycosylated–acylated flavonoids (ISG, IFG; HSG, HFG) followed the light-induced response of their glycosylated counterparts (SAP or LUT), particularly under blue light. This indicates that these compounds may have similar functions concerning photoprotection—substitution by hydroxycinnamic acids (HCAs) could affect their AOX as well as their transport and cellular localization [71]. On the other hand, their content may rise due to the higher availability of SAP and LUT, which may serve as substrates for acylation. FQA was the most abundant representative of soluble HCA derivatives present in barley leaves. FQA content exhibited a strong dependence on the irradiance of blue light, i.e., in examined irradiance range, it was observed only in blue treatments and WH. Although some HCAs are relatively strong antioxidants and UV-B attenuators, the observed increase in soluble FQA content might not be necessarily related to the production of protective metabolites. Alternatively, it may be the consequence of reduced longitudinal growth under blue light (or stronger white light). HCAs are incorporated into the cell walls during growth [72,73,74], thus reducing longitudinal growth together with overall activation of the PheC synthesis pathway, including its early steps (represented by the activation of PAL; Figure 8B), especially in BH treatment, may lead to the observed steep increase in FQA content.

Our results clearly showed that higher irradiance of blue light serves as an important environmental cue negatively affecting the ratio of B-mono/-di hydroxylated flavonoids (specifically ratio of isovitexin/homoorientin based flavonoids) in barley leaf tissue (Figure 5H). The number of hydroxyl groups on the B ring is the primary determinant of flavonoid antioxidant activity [75]. Such change in favor of B-dihydroxylated compounds is often considered as the plant enhancing tolerance against (light-induced) oxidative stress and damage (as discussed below) and as part of high-light acclimation [76]. This phenomenon is at least partially controlled at the GE level since the expression of the gene related to the F3′H enzyme (which catalyzes the formation of catechol group at flavonoid B-cycle) remains significantly upregulated in BH condition, even at the end of the acclimation phase (Figure 8C). This indicates that homoorientin derivatives are still synthesized; therefore, their content does not reach a final stable state two weeks after the plants were exposed to BH conditions. This is contrary to WH conditions, where the content of homoorientin derivatives is also higher (compared to RH treatment) but the *F3*′*H* expression is very low. Thus, the *F3*′*H* gene exhibits striking sensitivity to co-acting facets of light—irradiance (HI) and spectral quality (B). Although its response to HI itself (e.g., in WH, RH, GH conditions) appears to be negligible after prolonged acclimation to increased irradiance. The relative fold change of *F3*′*H* GE induced by higher irradiance of blue light was at least 3× higher compared to *CHS* and *PAL* genes (Figure 8C vs. A,B).

In summary, our data reveal that the production of all studied PheCs is positively regulated by higher irradiance of blue light. Production of isovitexin derivatives is mildly enhanced regardless of spectral quality while homoorientin derivatives require the presence of blue light in the spectrum, probably due to the high sensitivity of *F3*′*H* expression to this spectral component.

Such discrepancies between activation of GE related to PheC biosynthesis and the actual content of PheCs observed in plants exposed to different PAR irradiance and spectral quality might originate from different dynamics of gene expression and PheC metabolism—while the GE usually responds immediately, the detectable PheC accumulation occurs over hours or days. Further, contrary to GE, the content of PheCs remains relatively stable once synthesized, even after the removal of the inductive environmental cue [77]. Thus, if a high PheC content is observed but the expression of involved gene(s) is low, this could mean that we observe the final metabolic acclimation to current conditions while the initial GE signal already faded out.

### 3.4. Light as a Factor Affecting Plant (Photo-)Tolerance through Regulation of PheC Metabolism and AOX Enzymes

In this study, we observed a clear trend of soluble PheCs accumulating with increasing blue and white irradiance. Production of B-dihydroxylated flavonoids (homoorientin derivatives) was particularly enhanced. This response was not present or was markedly reduced during acclimation of plants to HI of other spectral qualities (G, R). Since the majority of flavonoids are effective UV absorbers, it could be concluded that blue light is an important component of natural sunlight which can coact with UV or even act independently (in artificial conditions) as a positive regulator of plant UV tolerance [57,78].

Moreover, acclimation of barley plants to increased blue and white irradiance markedly enhanced the in-vitro-measured AOX of leaf extracts (Figure 7). Contrary to the epidermal UV-A shielding, a positive effect of blue light on the AOX was detected even at the lowest irradiance of blue light. In addition, increasing irradiance of blue (and to a lesser extent also white) light enhanced the AOX activity of leaf extracts more pronouncedly than the efficiency of UV-A screening (Figure 6 vs. Figure 7 and Supplementary Figure S1 vs. S4). On the other hand, increasing irradiance of red light stimulated the efficiency of UV-A shielding more than the AOX activity of soluble PheCs. This could be due to red light being a reliable signal for top-of-canopy position, making UV shielding of greater importance than antioxidative function [79]. However, we presume that the observed increase in AOX is at least partially caused by the significantly higher proportion of B-hydroxylated flavonoids (such as LUT, HFG, HSG) in extracts (Figure 5H) (AOX shows a higher correlation and dependency on the content of homoorientin derivatives, Supplementary Figure S2 vs. Figure S3) and hypothetically also due to higher content of FQA. The dominant contribution of blue light to the AOX capacity of PheCs may also be related to their localization since high PAR selectively accumulates PheCs in mesophyll cells, where they contribute to AOX rather than to UV-A shielding [18]. Nevertheless, the possible presence of other LMWA with strong antioxidant activity in extracts cannot be excluded. Interestingly, in plant organs most exposed to UV-B radiation, the synthesis of HCA derivatives declines in favor of flavonoids [80,81], even though HCAs absorb UV-B more efficiently than flavonoids [82] and accumulation of HCAs is associated with higher tolerance of oxidative stress among barley genotypes [83]. As shown in Arabidopsis, acylation of some PheCs (such as saiginols) may enhance their UV-B absorption properties, which confers a fitness advantage to plants that produce them following exposure to prolonged UV-B [84,85]. However, whether the further binding of HCAs on flavonoid molecules (HFG, HSG) substantially affects (enhances) flavonoid AOX or shielding activity and their cellular localization compared to nonacylated counterparts is yet not clear.

Enhanced AOX may not only increase the tolerance of plants against UV-B stress, which is accompanied by overproduction of ROS in the chloroplast and especially in UV-B damaged PSII [86,87], but it can also alleviate the detrimental effects of excessive PAR stress, which is more frequent in natural environments (compared to UV-B stress). As with UV-B stress, excessive PAR leads to a significant production of ROS in the chloroplast (primarily singlet oxygen), however, their quality and origin within photosynthetic apparatus are different than in the case of UV-B stress. As shown by Agati et al. (2012) [88] B-dihydroxylated flavonoids were detected in chloroplasts in the proximity of ROS production sites. Thus, B-dihydroxylated flavonoids may participate in the reduction of ROS-induced damage and influence ROS-related signaling from the chloroplast [76,82,88]. In this context, the activation of phenylpropanoid and flavonoid pathways followed by the accumulation PheCs in leaf tissues could be classified as one of the many blue-light-induced acclimation responses of plants to high/excessive PAR. Since PheCs are relatively stable in leaf tissues [77], blue light could be used as an instrument for priming the plants against photo-oxidative stress [26] and should be further studied as a factor which increases plant tolerance against other environmental stresses interconnected with high ROS production (i.e., cross-tolerance) [89]. The importance of PheCs increases in conditions impairing the function of AOX enzymes (strong UV, high or low temperature, heavy metals) which are more sensitive to degradation/inactivation due to their protein nature.

Within this study, we also examined the regulation of the AOX enzymes *SOD* and *APX* at the level of GE, which increased proportionally to the irradiance of blue, white, and partially also green light (in the case of *SOD*; Figure 8D). Although the sensitivity of response (GE fold change) to blue light was much lower compared to GE of PheC related genes (especially *F3*′*H*), we can presume that enhanced SOD and APX activity may lead to reduced concentration of superoxide anion radicals and hydrogen peroxide under blue light conditions. Surprisingly, we observed the opposite trend (i.e., decrease in GE) with increasing irradiance of red light, which is in agreement with the decreased activity of SOD enzyme observed in *Boehmeria nivea* under red light [90]. This decrease may be due to the lack of a blue-light-induced CRY signal, which is part of high-light acclimation. Alternatively, this could indicate some sort of red-light-induced negative feedback, hypothetically involving phytochromes. The straightforward explanation of the lower production of ROS—and thus, lower demand for AOX enzymes—is not probable since the GE decreases with irradiance. A trend similar to the AOX enzymes—decreasing GE with increased red light irradiation—was observed for *SAG* (senescence associated gene 12), which is linked with natural and induced senescence in Arabidopsis [45] and barley [47,91]. This indicates that spectral quality and irradiance can affect leaf senescence, which is consistent with the current literature [92]. Together, these results indicate that blue-light-induced HI acclimatory responses—including accumulation of PheCs with antioxidant function as well as activation of enzymatic ROS scavenging machinery—are accompanied by a speeding-up of leaf ontogeny, whereas the absence of these responses at red light of the same irradiance alleviates the onset of senescence.

### 3.5. Spectral Quality Affects Expression of Genes Related to the PheCs Biosynthesis

As mentioned above, RT-qPCR analysis confirmed that barley plants acclimated to blue light had strongly upregulated GE for the production of PheC precursors (*PAL*, Figure 8A), as well as the “early genes” of PheC biosynthesis (*CHS* and *F3*′*H*, Figure 8B,C)—for a discussion of metabolic and physiological relevance, see Section 3.3 and Section 3.4. The stimulative effect of blue light on PheC-related GE has been already reported, e.g., for *Glycine max* seedlings [62], *Fagopyrum tataricum* sprouts [93], *Cyclocarya paliurus* [94], and *Agastache rugosa* plants [95]. However, in comparison with previous publications, our analyses proved for the first time that the degree of upregulation of PAL, CHS, and particularly *F3*′*H* in spring barley leaves depends strongly on blue light irradiance.

RNA-seq analysis confirmed that the “early genes” related to the flavonoid pathway (*CHS1*, *CHS/SSfp*, *CH/SSfp*, *CHIfp*, *C/Fifp*) were upregulated under the BH conditions in comparison with the GH-, RH-, and BL- (*CH/SSfp* was in this comparison insignificant) acclimated seedlings. In addition, we also observed increased expression of genes linked to the shikimic pathway (*DAHP*, *ESPS*, *CS*) under BH conditions compared to GH (*DAHP*, *ESPS*, *CS*), RH (*DAHP*, *CS*), or BL (*DAHP*, *CS*) light conditions (Figure 9). To our knowledge, the impact of spectral quality on the expression of genes linked to the shikimic pathway has not been reported. Additonally, the *ADT* (arogenate dehydratase) gene (linked to the biosynthesis of aromatic amino acids) seems to be upregulated under the BH conditions compared to the GH and RH acclimated plants (but not compared to BL plants). This phenomenon has not yet been described at the level of GE in the context of spectral quality before now. Interestingly, the “early genes” displayed the greatest differences in LogFC values, while none of the “late genes” related to the PheC biosynthesis displayed statistically significant overexpression in BH conditions compared to GH, RH, and BL treatments. The question is whether this result is caused by the different regulation of “early genes” and “late genes” in PheC biosynthetic machinery, (see Figure 2) or by possible epigenetic regulations. To address this question, direct experiments on the epigenetic regulations of the “late gene” group are needed.

### 3.6. Complex Role of miRNAs in the Regulation of PheCs Related Genes

**Figure 10:**
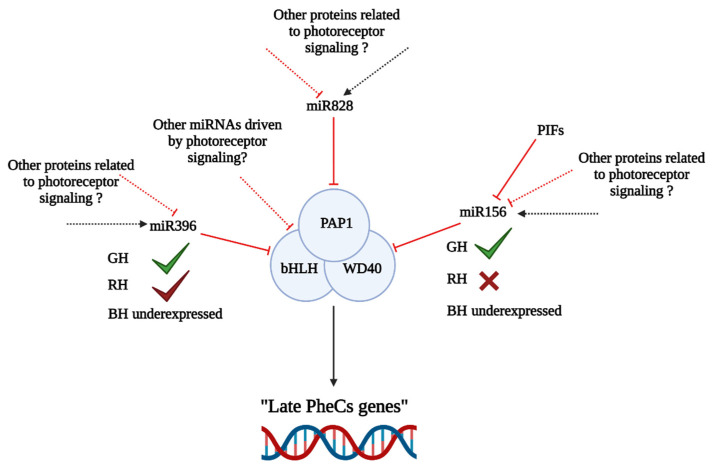
Schematic representation of possible effects of miRNAs on the MBW complex, which is responsible for the regulation of “late PheC genes”—presence (check), absence (cross), or downregulation of corresponding miRNAs under specific light conditions are indicated; full lines were validated by articles cited in the text above; dashed lines indicate possible alternative ways of regulation. Solid lines represent known interactions, whereas dashed lines indicate presumed alternative methods of regulation (not yet confirmed) (black—induction/upregulation; red—inhibition/downregulation). BH (blue), RH (red), and GH (green) light of high intensity; 400 µmol m^−2^ s^−1^.

In general, miRNAs are involved in PTGS and play an important role in regulating physiological processes, including plant development [96,97]. Three miRNAs seem to be essential (Figure 10) for PheC “late genes” regulation via MBW complex transcript degradation (PTGS). These miRNAs are: miR828, involved in the PAP1 (TF enhancing expression of PheC related genes) transcript degradation [98,99]; miR156, which induces the PTGS of the WD40 mRNA (this protein is necessary for MBW complex assembly); and miR396, which degrades the mRNA of bHLH74 in Arabidopsis [100]. However, the interaction of miRNAs with other transcripts encoding the bHLH proteins (proteins necessary for the MBW complex constitution) has not yet been reported, although such interaction could be expected—members of the same bHLH subfamily share structural similarity and expression patterns [101].

Our RNA-seq analysis revealed under expressed miRNAs (Table 1) in barley seedlings acclimated to BH conditions (compared to GH or RH)—specifically miR156 (compared to GH conditions) and miR396 (compared to both GH and RH conditions), which can degrade two out of three MBW mRNA transcripts via PTGS. This could explain why the “late genes” are not expressed under RH and GH. However, this does not explain why the “late genes” leading to anthocyanin biosynthesis are not transcribed in the BH. Even low temperature conditions, which strongly stimulate anthocyanin production in Arabidopsis, hardly affect barley anthocyanin accumulation (unpublished data). The question is whether this difference is due to primary DNA structure caused by epigenetic modification (e.g., DNA methylation) or a direct result of PTGS ensured by miRNAs. Epigenetic modifications could explain the lower responsiveness of “late genes” in barley compared to Arabidopsis, while the activity of miRNAs could explain the different responsiveness between “early genes” and “late genes” in barley under different experimental conditions. To validate such hypotheses, further study of the barley epigenome is needed.

In Arabidopsis, the connection between miR156 and R/FR (red/far-red) signaling via PhyB and PIFs has been documented [102]. Expression of miR156 seems to be linked to the R/FR signaling cascade, but experimental data focused on miR156 expression in plants acclimated to monochromatic red light conditions are missing (for blue and green monochromatic lights as well). Similarly, miR396 expression patterns under the various monochromatic light conditions have not been reported for barley. However, for other plant species, the impact of light stress on miRNA profile has been described. In *Zea mays* [103], *Populus tremula* [104], and *Prunus persica* [105], changes in miRNA profile were observed after UV-B application. Similarly, changes in the miRNA profile of *Dendrocalamus latiflorus* seedlings after exposure to white light [106] were documented. FR-responsive miRNAs were described in *Glycine max* [107] targeting genes related to PheC metabolism. This indicates that spectral quality induced changes in miRNA profiles can affect PheC related genes and further strengthens our hypothesis about the monochromatic light-responsive miRNAs outlined in Figure 10.

Ultimately, we propose that several miRNAs can affect GE of PheC related genes (mainly the “late genes”), but to confirm this hypothesis, additional experiments focused on sequencing small RNAs in the same experimental conditions are needed. The validity of our statements with regards to the influence of different irradiances of monochromatic light on levels of miRNAs in spring barley should be confirmed or refuted in subsequent studies, bearing in mind that library preparation for RNA-seq analysis, as well as the total RNA isolation procedure itself, can affect the acquired data. Deeper research should also include comparative analysis of miRNAs under various monochromatic light conditions in barley, and it should further search for the possible miRNA targets (mRNAs). Both wet-lab and in silico approaches should be considered in the future to confirm current and also to search for novel miRNA targets in spring barley and shed light on the mysterious and overlooked level of GE regulation of LMWAs, including PheCs.

## 4. Materials and Methods

### 4.1. Cultivation of Plant Material

Seeds of spring barley *Hordeum vulgare* L. cv Bojos were sown into square pots (a = 13 cm, V = 2200 cm^3^) containing a mixture (1:1 *v*/*v*) for house plants and gardening substrate (Agro CS, Česká Skalice, Czech Republic). Substrate was kept well-watered without additional fertilizers. Plants were cultivated in the growth chamber Phytoscope FS130 (PSI, Drásov, Czech Republic) with a 16-hour light period at 22 °C followed by an 8-hour dark period at 20 °C. Air humidity was approx. 60%. To ensure normal development of plants during germination, barley was grown for one week under low irradiation white light (RGB 1:1:1; total irradiance 100 μmol m^−2^ s^−1^). Subsequently, plants were acclimated to various combinations of irradiances (L—low 100, M—medium 200, or H—high 400 µmol m^−2^ s^−1^) and spectral qualities (R—red, G—green, B—blue, or W—white containing R:G:B in a ratio of 1:1:1) for 7 days. Table 2 summarizes spectral conditions and designation of treatments. The LED spectral properties used in the experiment are shown in Supplementary Figure S6. Samples of barley secondary leaves (central segments) were collected on day 14. Sampling procedure and sample preparation is further specified in the following sections. The samples were immediately frozen in liquid nitrogen and stored at −80 °C till analysis.

### 4.2. Epidermal UV-A Shielding

In vivo measurement of epidermal UV-A shielding was performed after 14 days of growth using DUALEX^®^ Leaf Analyser (ForceA, Orsay, France). Epidermal UV-A shielding was measured at the central part of barley secondary leaves (12 samples per light treatment) approximately 1 h before the start of the light phase. After the measurement, leaves were used for the extraction of soluble PheCs (see below).

### 4.3. Extraction of Soluble Phenolic Compounds

Plant extracts of soluble PheCs were prepared from central segments of *Hordeum vulgare* secondary leaves (100 ± 5 mg of fresh weight (FW), approximately 2 segments). Six samples per treatment were collected from dark-acclimated plants one hour before the light phase. Samples were homogenize using mortar and pestle in 3 mL of 40% methanol (CH_4_O, ≥99.9%, Mr = 32.04 g·mol^−1^, Sigma-Aldrich, Schnelldorf, Germany) with a small amount of sea sand (Penta, Prague, Czech Republic). The homogenate was ultrasonified for 5 min (K-51E, Kraintek Czech s.r.o., Hradec Králové, Czech Republic) and centrifuged (EBA 20, Hettich Zentrifugen, Tuttlingen, Germany) at 6000 rpm for 3 min. The supernatant was made up to 3 mL with 40% methanol in a volumetric tube and filtered into amber vials using a syringe with a Spartan filter (13/0.2 RC, Whatman, Dassel, Germany). Prepared extracts of PheCs were stored at −22 °C till analysis.

### 4.4. HPLC-DAD Based Quantification of Soluble Phenolic Compounds

The semiquantitative analysis of soluble PheCs (extracted in 40% methanol as described above) was performed on an Agilent 1200 HPLC system (Agilent Technologies, Santa Clara, CA, USA) equipped with a UV-VIS absorption diode array detector (DAD; G1315D; Agilent Technologies, Santa Clara, CA, USA). PheCs were separated using Hypersil Gold chromatographic column (C18, 50 mm × 2.1 mm, 1.9 μm, Thermo Scientific, Waltham, NJ, USA). The column was tempered at 30 °C during the whole separation process. Two acidified acetonitrile–water solutions were used as the mobile phases (m.p. A—5% ACN; m.p. B—80% ACN; C_2_H_3_N, ≥99.9%, Mr = 41.05 g·mol^−1^, Sigma-Aldrich, Schnelldorf, Germany). The mobile phase was acidified by formic acid (CH_2_O_2_, Mr = 46.03 g·mol^−1^, Sigma-Aldrich, Schnelldorf, Germany) in a ratio of 999:1, *v*/*v*. The flow of mobile phases was set to 0.3 mL/min^−1^. The gradient of mobile phases is shown in Table 3.

Samples were injected in a volume of 10 µL. The relative quantity of PheCs was determined by manual integration of peaks detected at 314 nm. The peak area was further adjusted to the FW of the sample). Retention times and UV–Vis absorption spectra were acquired to facilitate compound identification and alignment with HPLC-DAD-MS data, which were primarily used for compound identification (Table 3). The total content of soluble PheCs for each chromatogram was evaluated as the sum of peak areas adjusted to the FW of each sample.

### 4.5. The Identification of Soluble Phenolic Compounds

The qualitative analysis of PheCs present in spring barely secondary leaves was performed using a UHPLC-DAD system (UltiMate 3000, Dionex, Sunnyvale, CA, USA) in tandem with a (Q-TOF) mass spectrometer micrOTOF-QII (Bruker Daltonics, Bremen, Germany). For the separation of PheCs, the same procedure as HPLC-DAD quantitative analysis was applied. The mass spectrometry analysis was conducted in negative ion mode—the electrospray was used as the ion source (end plate offset = 500 V, capillary voltage = 2900 V, nebulizer pressure 3.5 Bar, dry gas flow = 10 L min^−1^, temperature = 200 °C). The mass spectra were acquired in the range of 50 to 1500 *m*/*z*. Furthermore, collision-induced dissociation of detected compounds (at 35 eV) was performed to obtain MS (fragmentation) spectra. The tentative identification of PheCs was conducted based on the assessment of their retention behavior and by comparison of their UV–Vis absorption spectra, exact *m*/*z*, and fragmentation patterns with the literature [108,109,110], as well as with corresponding commercially available standards (saponarin; Extrasynthése, FR) or similar compounds (such as homoorientin, isovitexin, luteolin, apigenin, and ferulic acid; Extrasynthése, Genay, Fance) (Supplementary Section S1: The identification of soluble phenolic compounds).

### 4.6. Antioxidant Activity Assay

The antioxidant activity of PheC extracts was determined by colorimetric assay using the stable DPPH^•^ radical (2,2-Diphenyl-1-picrylhydrazyl, Sigma-Aldrich, Schnelldorf, Germany, Cat No. 1898-66-4). The DPPH solution used for measurement (and calibration) was prepared by the dissolution of 0.01875 g of DPPH^•^ in 250 mL of 100% methanol. The method was calibrated using the antioxidant Trolox ((±)-6-Hydroxy-2,5,7,8-tetramethylchromane-2-carboxylic acid, Calbiochem, Cat No. 53188-07-1). The set of calibration solutions was prepared by the dilution of Trolox stock solution (1 mM; 0.0125 g in 50 mL of 100% methanol) to final concentrations of 0, 25, 50, 100, 200 and 300 µM. For the measurement of antioxidant activity, 2 mL of DPPH solution was mixed with 0.5 mL of Trolox solution (calibration samples), 0.5 mL of methanol (blank samples), or 0.5 mL of PheC-containing plant extracts. The mixture was incubated for 10 min in the dark before the spectrophotometrical analysis. The relative change of sample absorbance at 515 nm (compared to the blank) was recorded using a double-beam absorption spectrophotometer (Specord 250, Analytik Jena, Jena, Germany) with a monochromator width slit set to 0.5 nm. The resulting values were expressed as Trolox-equivalent antioxidant capacity (TEAC) based on established calibration curves and further adjusted to the FW of samples.

### 4.7. RNA Isolation, DNAse Treatment and Reverse Transcription

Central leaf segments (approx. 50 mg) were sampled (three biological replicates per treatment) and immediately frozen in liquid nitrogen. Frozen plant tissue was homogenized by mortar and pestle, washed with 0.5 mL of TRIzol (Sigma-Aldrich, St. Louis, MO, USA, Cat No. T9424) in a fresh Eppendorf tube, and stored in the freezer at −80 °C until RNA isolation. Total RNA extraction was performed according to the manufacturer’s instructions. TURBO DNA-free protocol was used (Ambion, Austin, TX, USA, Cat No. AM 1907) to remove DNA traces in samples. The quality and quantity of RNA were assessed using a NanoPhotometer (Implen, Westlake Village, CA, USA). For a random subset of samples, the RNA integrity was also determined by 1.5%-denaturing agarose gel electrophoresis. An amount of 1 µg of total RNA per sample was taken for the reverse transcription using a First Strand cDNA kit (Thermo Scientific, Waltham, NJ, USA, Cat No. K1612).

### 4.8. qPCR

Primer sequences (Supplementary Table S3, Thermo Fisher Scientific, Waltham, NJ, USA) were selected based on the literature [111,112,113,114,115,116], supplied by the Thermo Fisher Scientific, and diluted according to the manufacturer’s instructions using nuclease free water. For each qPCR reaction, 5 µL of EliZymeGreen MIX AddROX (Elisabeth Pharmacon, Brno, Czech Republic, Cat No. EZ4614), 1 µL of diluted forward, 1 µL of diluted reverse primers (to adjust a final concentration of oligonucleotides in the reaction mixture to 250 nM), and 2 µL of nuclease-free water were used. Diluted cDNA (1 µL; 20 µL cDNA:20 µL nuclease-free water) was added to the mixture. qPCR conditions for all listed primer pairs were the same: 1 min of initial denaturation (95 °C) followed by 35 cycles of 95 °C for 15 s, 57 °C for 15 s, and 72 °C for 15 s in 96-well optical reaction plates. Subsequently, a melting curve analysis was performed. All qPCR reactions were performed at Roche LC480^®^ Instrument (Roche Diagnostics GmbH, Mannheim, Germany) in 3 technical replicates. RT-qPCR data were processed and normalized as described in Livak and Schmittgen (2001) [117]. Alpha tubulin was chosen as a reference gene. Differential expression was calculated relative to WM (white light medium irradiance).

### 4.9. RNA Sequencing and Transcriptome Analysis

Total RNA for transcriptome sequencing was isolated and DNase treated as described above (Section 4.7). Purified RNA from 3 biological replicates was measured with a NanoPhotometer (Implen, Westlake Village, CA, USA) and pooled together in the same amount (approximately 6 µg from each sample per treatment). After preparation, samples were frozen in liquid nitrogen, and transported on dry ice to the Novogene company (Stockton, Sacramento, CA, USA). A quality check via bioanalyzer followed (all samples had RNA integrity number/RIN/above 6) and sequencing was performed using the Illumina HiSeq platform. Raw sequencing data were uploaded to Galaxy webserver [118] and trimmed and processed using the Kallisto quant tool (Freiburg, Germany, tool version 0.46.0, *Hordeum vulgare* IBC PGSB v2 built in transcriptome was applied as a reference accessed on 20 April 2020). The resulting tables of transcript abundances were exported and merged to a .txt file and processed using edgeR workflow (Supplementary Section S2: EdgeR workflow code) [119]. Genes with significantly different expression rates (DEGs) with false discovery rates (FDR) below 0.1 were uploaded to PLAZA Monocots 4.0 portal (https://bioinformatics.psb.ugent.be/plaza/versions/plaza_v4_monocots/, accessed on 11 June 2021) [120] and gene IDs were exported and processed using STRINGdb (https://string-db.org/, accessed on 6 October 2021) with parameters set as follows: minimum required interaction score of 0.400, 10 maximum interactions to show, disabled structure previews in bubbles, hidden disconnected nodes, shown input protein names and k-means clustering. All genes annotated at STRINGdb related to PheC biosynthesis were exported for further processing, including their abundances. Results focused on PheC related metabolism are listed in Figure 8.

### 4.10. Data Visualisation and Statistical Analysis

Statistical testing and data visualization were performed in GraphPad Prism (v9.1.2.226, GraphPad Software, San Diego, CA, USA) and RStudio (v1.3.1093, RStudio Inc., Boston, MA, USA). Homoskedasticity and data normality for further analysis by parametric tests were verified by residuals vs. fitted plot and QQ plot followed by Leven and Shapiro-Wilk test. After verifying the variance and normal distribution, a two-way ANOVA (analysis of variance) was used to assess the effect of spectral quality, irradiation, their interaction on PheC content, related sample parameters (epidermal UV-A shielding, AOX, etc.), and qPCR data. For multiple group comparisons, Tukey’s post hoc test was applied.

Further cluster analysis and heatmaps illustrating the differences in the relative content of PheCs across treatments differing in spectral compositions and irradiance were created (RStudio, Supplementary Section S3). The relative contents of each PheC per treatment were averaged (*n* = 5–6) and subsequently normalized to the maximum value among all light treatments. Cluster analysis was performed on normalized PheC quantitative data based on average linkage. Distance between rows (spectral conditions) and columns (PheCs) was determined by the Euclidean distance.

## 5. Conclusions

We present evidence that the blue spectral component of HI is essential for the accumulation of PheCs and activation of enzymatic ROS scavenging machinery (such as SOD, APX) in secondary leaves of *Hordeum vulgare*. Intriguingly, the changes in PheC metabolic profile (their relative content and quantity) enhanced the AOX of leaf extracts considerably more than the UV-A shielding properties of leaves. Specifically, it could be attributed to pronounced biosynthesis of dihydroxylated PheCs (homoorientin derivatives), which are at least partially caused by GE of F3′H under high blue light irradiance. Such responses were not induced by other spectral treatments, regardless of the examined irradiance range (100, 200, or 400 μmol m^−2^ s^−1^). Our data also suggests that spectral quality could affect miRNAs involved in the PTGS of PheC-related genes. Three miRNAs (156, 828, and 396) seem to mediate the degradation of MBW complex transcripts, which could explain the different responsiveness between the expression of “early genes” and “late genes” in barley under different light treatments. On the other hand, more experiments focused on the barley epigenome under monochromatic light conditions are needed to explain the extent to which miRNAs affect PheC biosynthesis and to completely understand the complex epigenetic regulatory mechanisms leading to PheC biosynthesis, apart from miRNAs.

## Figures and Tables

**Table 1:** Analysis of miRNAs with the potential to induce PTGS which were underexpressed under BH conditions compared to GH, RH, and BL, respectively.

Comparison	Plaza ID	miRNA
Compared to GH	HVU0042G1661	miR396
	HVU0042G2193	miR156
	HVU0038G1160	miR1122
	HVU0038G1161	miR1122
	HVU0040G1583	miR1122
	HVU0040G1584	miR1122
Compared to RH	HVU0042G1661	miR396
	HVU0045G0592	miR169_5
	HVU0038G1160	miR1122
	HVU0038G1161	miR1122
Compared to BL	HVU0037G2782	miR169_5

**Table 2:** Specification of light treatments (i.e., irradiance and spectral quality) used during the 7-day acclimation period of spring barley plants (*Hordeum vulgare* L. cv Bojos). W (white light), B (blue light), R (red light), G (green light), L (low irradiance, 100 μmol m^−2^ s^−1^), M (medium irradiance, 200 μmol m^−2^ s^−1^), and H (high irradiance, 400 μmol m^2^ s^−1^).

PAR Irradiance [μmol m^−2^ s^−1^]	Spectrum in PAR Region	Group ID
100	R	RL
200	R	RM
400	R	RH
100	G	GL
200	G	GM
400	G	GH
100	B	BL
200	B	BM
400	B	BH
100	W	WL
200	W	WM
400	W	WH

**Table 3:** Gradient of mobile phases used for HPLC based PheC separation.

Time [min]	A: 5% ACN [%]	B: 80% ACN [%]
0	100	0
2	95	5
10	80	20
15	60	40
18	20	80
22	0	100
24	0	100

## Data Availability

Processed and derived data are available from the corresponding authors V.Š. and J.N. on request.

## Supplementary Materials

The following supporting information can be downloaded at: https://www.mdpi.com/article/10.3390/ijms23126533/s1.
