# Supplementary material for: Regulation of Phenolic Compound Production by Light Varying in Spectral Quality and Total Irradiance
Source: Int J Mol Sci. 2022 Jun 10;23(12):6533. doi: 10.3390/ijms23126533 (PMC9223736; doi:10.3390/ijms23126533)
Supplement: Supplementary file 1 [file ijms-23-06533-s001.zip › ijms-1745529-supplementary.pdf]

Supplementary material

Regulation of Phenolic Compound Production by Light Varying in Spectral Quality and Total Irradiance

Radomír Pech [1,†], Adriana Volná [1,†], Lena Hunt [2], Martin Bartas [3], Jiří Červeň [3], Petr Pečinka [3], Vladimír Špunda [1,4,*] and Jakub Nezval [1,*]

[1] Department of Physics, Faculty of Science, University of Ostrava, 710 00 Ostrava, Czech Republic; radomir.pech@osu.cz (R.P.); adriana.volna@osu.cz (A.V.)
[2] Department of Experimental Plant Biology, Faculty of Science, Charles University, 128 00 Praha, Czech Republic; huntl@natur.cuni.cz
[3] Department of Biology and Ecology, Faculty of Science, University of Ostrava, 710 00 Ostrava, Czech Republic; martin.bartas@osu.cz (M.B.); jiri.cerven@osu.cz (J.Č.); petr.pecinka@osu.cz (P.P.)
[4] Global Change Research Institute, Czech Academy of Sciences, 603 00 Brno, Czech Republic
* Correspondence: vladimir.spunda@osu.cz (V.Š.); jakub.nezval@osu.cz (J.N.)
† These authors contributed equally to this work.

Content

1. Identification of soluble phenolic compounds 3

2. EdgeR workflow code 4

3. Pheatmap workflow code 5

Table S1: Summary of Two-Way ANOVA main results – total PheCs content, relative content of individual PheCs, epidermal UV-A shielding, and antioxidant capacity. 6

Table S2: Summary of Two-Way ANOVA main results – Gene expression. 7

Table S3: Gene expression – Primers. 8

Table S4: Relative content of individual soluble PheCs and total PheCs content determined in various light treatments 9

Table S5: Main results of HPLC-DAD-MS analysis used for tentative identification of detected PheCs. 10

Figure S1: Correlation between total soluble PheCs content in leaf extract and its TEAC (Trolox Equivalent Antioxidant Capacity 11

Figure S2: Correlation between content of isovitexin derivatives detected in leaf extracts and TEAC (Trolox Equivalent Antioxidant Capacity 12

Figure S3: Correlation between content of homoorientin derivatives detected in leaf extracts and TEAC (Trolox Equivalent Antioxidant Capacity 13

Figure S4: Correlation between content of total soluble PheCs detected in leaf extract and epidermal UV-A shielding. 14

Figure S5: Typical chromatograms of separated phenolic compounds as obtain from HPLC-DAD analysis. Blue line (high intensity blue light treatment), red line (high intensity red light treatment). FQA (feruloylquinic acid), LUT (lutonarin), SAP (saponarin), ISC (isoscoparin derivative), HSG (homoorientin-7-O-[6-sinp]-glc), HFG (homoorientin-7-O-[6-fer]-glc), ISG (isovitexin-7-O-[6-sinp]-glc), IFG (isovitexin-7-O-[6-fer]-glc). 15

Figure S6: LEDs emission spectra measured approximately at the height of spring barely secondary leaves in growth chamber 16

References 17

1. Identification of soluble phenolic compounds

The plant extracts obtained from barley secondary leaves contained significant amounts of PheCs mainly flavonoids (specifically flavones) and hydroxycinnamic acid derivatives (HCA). The summary of spectral (UV-VIS absorption, MS and MS^2) and elution (retention times; Rt) properties used for the tentative identification is shown in Table S5. The most abundant HCA as well as the only one belonging to this class which was quantified within this study was detected at Rt 3.9 min. It exhibited absorption spectrum very similar to ferulic acid with the main maximum at 323 nm (Table S5). This compound (m/z $[M-H]^-$ 367) was tentatively identified as feruloyl-quinic acid (FQA). The identity of this compound was supported by the fragments 193 and 191 which could be assigned to ferulic and quinic acid parts of the molecule respectively. Same fragments were previously reported for feruloyl quinic acids e.g by [1], [2]. However, fragment 193 was observed only for 1-FQA, 3-FQA, 4-FQA isomers whereas 191 for 5-FQA [2] suggesting the possible co-elution of more than one FQA isomer. Alternatively, it might be a consequence of different fragmentation conditions used in this study (compared to [2]). The separation continued by the elution of two di-glycosylated flavones (Rt = 6.9 min, Rt = 8.2 min). The compound eluted at 8.2 min was conclusively identified as the saponarin based on the comparison of spectral and elution properties with commercially available standard. The second eluted compound (Rt = 6.9 min) exhibited absorption spectrum typical for luteolin based flavonoid (255, 269, 348 nm), shorter retention time compared to luteolin (aglycone) and homoorientin (luteolin-glucoside) as well as higher molecular weight (m/z $[M-H]^-$ 609). Together with comparison of its fragmentation pattern (Table S5) with data published by [3] this compound was tentatively identified as lutonarin. Rather low-abundant flavonoid eluted after the saponarin (at 8.7 min) exhibited UV-VIS spectra similar to 225, 270, 345 nm luteolin derivatives, however the fragmentation of its pseudomolecular ion led to production of abundant fragment m/z 461 which is typical for isoscoparine derivatives (but also could be produced e.g. from luteolin based glycosylated-acylated compounds), further comparison of fragmentation data with the literature [3] led to the conclusion that this isoscoparine derivative is most probably isoscoparine-7-O-glucoside. This compound was followed by the two homoorientin (Rt = 10.3 and 10.6 min) and two isovitexin derivatives (Rt 11.3 and 11.8 min). Collision induced dissociation of pseudomolecular $[M-H]^-$ ions belonging to homoorientin derivatives led to production of characteristic fragment m/z 447, whilst isovitexin derivatives produced fragment m/z 431. The pair of isovitexin derivatives as well as the pair of homoorientin derivatives exhibited very similar fragmentation pattern but differed in the m/z of their pseudomolecular ions (Table S5). The two distinguishable neutral losses responsible for this difference were 368 resp 338 m.u. which could be interpreted as the loss of acyl-glucose moiety from to molecule, specifically sinapoyl-glucose resp. feruloyl-glucose [3]. Thus these 4 compounds were tentatively identified as acylated-glycosylated derivatives of homoorientin and isovitexin (Table S5).

2. EdgeR workflow code

```
if (!requireNamespace("BiocManager", quietly = TRUE))
install.packages("BiocManager")

BiocManager::install("limma")

if (!requireNamespace("BiocManager", quietly = TRUE))
install.packages("BiocManager")
BiocManager::install("edgeR")

library(edgeR)

x<- read.delim("KallistoRGB.txt",dec=",",row.names=1)
str(x)

y <- DGEList(counts=x,group=1:4)
bcv <- 0.1
y1 <- calcNormFactors(y)
plotMDS(y1)
et <- exactTest(y1, pair=c(3,2),dispersion=bcv^2)
plotMD(et,main="B400xG400")
out <- topTags(et, n=Inf)
write.csv(out, file="porovnaniBxG.csv")

et <- exactTest(y1, pair=c(4,2),dispersion=bcv^2)
plotMD(et,main="B400xR400")
out <- topTags(et, n=Inf)
write.csv(out, file="porovnaniBxR.csv")

et <- exactTest(y1, pair=c(1,2),dispersion=bcv^2)
plotMD(et,main="B400xB100")
out <- topTags(et, n=Inf)
write.csv(out, file="porovnaniB400xB100.csv")
```

3. Pheatmap workflow code

```
if (!require(pheatmap))
install.packages('pheatmap')

library(pheatmap)

x<-read.table("clipboard", header=T, dec=",")
x<-as.matrix(x)

pheatmap(
   x,
   cluster_rows = FALSE,
   clustering_distance_rows = "euclidean",
   clustering_distance_cols = "euclidean",
   cutree_rows = NA,
   cutree_cols = NA,
   treeheight_row = 50,
   treeheight_col = 40,
   border_color = "grey30",
   cellwidth = NA,
   cellheight = NA,
   scale = "column"
)
```

Table S1: Summary of Two-Way ANOVA main results – total PheCs content, relative content of individual PheCs, epidermal UV-A shielding, and antioxidant capacity.

**HPLC – Total content of PheCs**				
**Source of Variation**	**% of total variation**	**P value**	**P value summary**	**Significant?**
Interaction	14.21	<0.0001	****	Yes
Spectral quality	32.40	<0.0001	****	Yes
Irradiance	37.17	<0.0001	****	Yes
**Dualex – Epidermal UV-A shielding**				
**Source of Variation**	**% of total variation**	**P value**	**P value summary**	**Significant?**
Interaction	11.27	<0.0001	****	Yes
Spectral quality	12.90	<0.0001	****	Yes
Irradiance	40.63	<0.0001	****	Yes
**DPPH – Antioxidant capacity**				
**Source of Variation**	**% of total variation**	**P value**	**P value summary**	**Significant?**
Interaction	15.36	<0.0001	****	Yes
Spectral quality	50.55	<0.0001	****	Yes
Irradiance	24.69	<0.0001	****	Yes
**SAP (saponarin)**				
**Source of Variation**	**% of total variation**	**P value**	**P value summary**	**Significant?**
Interaction	14.52	<0.0001	****	Yes
Spectral quality	35.07	<0.0001	****	Yes
Irradiance	32.22	<0.0001	****	Yes
**LUT (lutonarin)**				
**Source of Variation**	**% of total variation**	**P value**	**P value summary**	**Significant?**
Interaction	25.08	<0.0001	****	Yes
Spectral quality	40.57	<0.0001	****	Yes
Irradiance	17.66	<0.0001	****	Yes
**ISG (isovitexin-7-O-[6-sinp]-glc)**				
**Source of Variation**	**% of total variation**	**P value**	**P value summary**	**Significant?**
Interaction	6.821	0.0031	**	Yes
Spectral quality	13.74	<0.0001	****	Yes
Irradiance	58.00	<0.0001	****	Yes
**HSG (homoorientin-7-O-[6-sinp]-glc)**				
**Source of Variation**	**% of total variation**	**P value**	**P value summary**	**Significant?**
Interaction	13.39	<0.0001	****	Yes
Spectral quality	26.39	<0.0001	****	Yes
Irradiance	36.56	<0.0001	****	Yes
**IFG (isovitexin-7-O-[6-fer]-glc)**				
**Source of Variation**	**% of total variation**	**P value**	**P value summary**	**Significant?**
Interaction	10.33	0.0002	***	Yes
Spectral quality	21.86	<0.0001	****	Yes
Irradiance	46.76	<0.0001	****	Yes
**HFG (homoorientin-7-O-[6-fer]-glc)**				
**Source of Variation**	**% of total variation**	**P value**	**P value summary**	**Significant?**
Interaction	23.54	<0.0001	****	Yes
Spectral quality	18.32	<0.0001	****	Yes
Irradiance	32.54	<0.0001	****	Yes

Table S2: Summary of Two-Way ANOVA main results – Gene expression.

**CHS (chalcone synthase)**				
**Source of Variation**	**% of total variation**	**P value**	**P value summary**	**Significant?**
Interaction	35.18	0.0013	**	Yes
Spectral quality	29.16	0.0004	***	Yes
Irradiance	9.267	0.027	*	Yes
**PAL (phenylalanine ammonia lyase)**				
**Source of Variation**	**% of total variation**	**P value**	**P value summary**	**Significant?**
Interaction	30.15	0.0006	***	Yes
Spectral quality	42.75	<0.0001	****	Yes
Irradiance	7.095	0.0262	*	Yes
**F3´H (flavonoid 3-hydroxylase)**				
**Source of Variation**	**% of total variation**	**P value**	**P value summary**	**Significant?**
Interaction	41.2	0.0019	**	Yes
Spectral quality	15.68	0.0234	*	Yes
Irradiance	10.05	0.0414	*	Yes
**SOD (superoxide dismutase)**				
**Source of Variation**	**% of total variation**	**P value**	**P value summary**	**Significant?**
Interaction	48.02	<0.0001	****	Yes
Spectral quality	18.02	0.0003	***	Yes
Irradiance	18.03	0.0001	***	Yes
**APX (ascorbate peroxidase)**				
**Source of Variation**	**% of total variation**	**P value**	**P value summary**	**Significant?**
Interaction	30.98	0.0147	*	Yes
Spectral quality	17.75	0.0216	*	Yes
Irradiance	14.62	0.0178	*	Yes
**SAG (senescence associated gene 12)**				
**Source of Variation**	**% of total variation**	**P value**	**P value summary**	**Significant?**
Interaction	47.85	<0.0001	****	Yes
Spectral quality	26.25	0.0002	***	Yes
Irradiance	4.716	0.0897	ns	No

Table S3: Gene expression – Primers.

Name	Primer sequence	Publication
Hv_CHS_Forward	CCGACTACCCGGACTACTAC	Han et al. 2016 [4]
Hv_CHS_Reverse	TGTACCTCTTCCTGATCTGCG	Han et al. 2016 [4]
Hv_PAL_Forward	GCAGCACAGCAAAACCGTATCTC	Ghannam et al. 2016 [5]
Hv_PAL_Reverse	ACCATTCCACTCCTTCAGGCAC	Ghannam et al. 2016 [5]
Hv_F3´H_Forward	GCCAGGGAGTTCAAGGACA	Shoeva et al. 2016 [6]
Hv_F3´H_Reverse	CTCGCTGATGAATCCGTCCA	Shoeva et al. 2016 [6]
Hv_αTUB_Forward	CCATCAAGACCAAGCGCACTA	Cai et al. 2018 [7]
Hv_αTUB_Reverse	CATACCCTCACCCACATACCA	Cai et al. 2018 [7]
Hv_SOD_Forward	CCGAAGATGAAATCCGCCAT	Shagimardanova et al. 2010 [8]
Hv_SOD_Reverse	CGGCCAATGATTGAATGTGG	Shagimardanova et al. 2010 [8]
Hv_APX_Forward	CGGAGCTTTTGAGTGGTGACA	Shagimardanova et al. 2010 [8]
Hv_APX_Reverse	CCGCAGCATATTTCTCCACAA	Shagimardanova et al. 2010 [8]
Hv_SAG_Forward	ACGAGGAGCGAGCTATCATT	Parrott et al. 2010 [9]
Hv_SAG_Reverse	GACCATTGTACACGCCATTC	Parrott et al. 2010 [9]

Table S4: Relative content of individual soluble PheCs and total PheCs content determined in various light treatments.

	FQA		LUT		SAP		ISD		HSG		HFG		ISG		IFG		SUM
**Treatment**	**Area / FW**	**% in total PheCs**	**Area / FW**	**% in total PheCs**	**Area / FW**	**% in total PheCs**	**Area / FW**	**% in total PheCs**	**Area / FW**	**% in total PheCs**	**Area / FW**	**% in total PheCs**	**Area / FW**	**% in total PheCs**	**Area / FW**	**% in total PheCs**	**Area / FW**
WL	0.00	0.00	90.54	1.66	3867.52	71.00	96.60	1.77	115.69	2.12	30.56	0.56	1006.73	18.48	239.38	4.39	5447.02
WM	0.00	0.00	114.19	1.43	5630.24	70.67	101.34	1.27	192.32	2.41	54.87	0.69	1536.60	19.29	337.40	4.23	7966.97
WH	240.04	1.71	587.68	4.18	9213.28	65.60	186.28	1.33	610.00	4.34	182.09	1.30	2477.82	17.64	547.28	3.90	14044.47
BL	323.33	4.99	147.08	2.27	4945.06	76.28	78.09	1.20	110.71	1.71	29.23	0.45	631.08	9.74	217.94	3.36	6482.52
BM	900.66	7.41	616.24	5.07	8336.33	68.55	234.99	1.93	335.61	2.76	98.19	0.81	1228.09	10.10	411.58	3.38	12161.69
BH	1334.75	7.16	1423.14	7.63	12236.78	65.62	371.49	1.99	622.14	3.34	246.13	1.32	1816.67	9.74	596.42	3.20	18647.53
GL	0.00	0.00	77.05	1.78	3372.07	77.87	162.84	3.76	33.74	0.78	32.15	0.74	463.37	10.70	189.42	4.37	4330.65
GM	0.00	0.00	86.22	1.41	4474.85	73.11	157.88	2.58	71.30	1.16	43.33	0.71	1045.94	17.09	241.29	3.94	6120.81
GH	0.00	0.00	164.50	1.84	6193.11	69.18	196.09	2.19	228.11	2.55	55.54	0.62	1777.79	19.89	336.55	3.76	8951.67
RL	0.00	0.00	49.58	0.86	4173.62	72.59	110.31	1.92	82.28	1.43	53.74	0.93	1065.60	18.53	214.81	3.74	5749.93
RM	0.00	0.00	81.96	1.44	4159.26	72.93	212.32	3.72	62.75	1.10	41.97	0.74	887.88	15.57	256.65	4.50	5702.79
RH	0.00	0.00	112.76	1.63	4405.12	63.72	286.24	4.14	149.65	2.16	64.27	0.93	1564.76	22.63	330.56	4.78	6913.36

Table S5: Main results of HPLC-DAD-MS analysis used for tentative identification of detected PheCs.

Abbreviation	Compound	Rt [min]	λmax [nm]	Characteristic ion m/z of $[M-H]^-$	Fragment ions m/z
FQA	Feruloyl quinic acid	3.9	240, 296sh, 323	367	134, 193, 191
LUT	Isoorientin-7-O-glc (Lutonarin)	6.9	255, 269, 348	609	447, 327, 357, 489*
SAP	Isovitexin-7-O-glc (Saponarin)	8.2	270, 336	593	431, 311, 473
ISD	Isoscoparine derivative (-7-O-glc most probably)	8.7	255, 270, 345	623	461, 341, 503, 608*, 327*
HSG	Isoorientin-7-O-[6-sinp]-glc	10.3	271, 340	815	447, 461, 327
HFG	Isoorientin-7-O-[6-fer]-glc	10.6	254, 272, 340	785	447, 461, 327
ISG	Isovitexin-7-O-[6-sinp]-glc	11.3	271, 334	799	431, 445, 311*, 473*, 341
IFG	Isovitexin-7-O-[6-fer]-glc	11.8	271, 333	769	431, 445, 311, 473*

FQA was identified based on the comparison of UV absorption. MS and MS^2 with results presented in Kuhnert et al. 2010 and Masike et al. 2017. Similarly all flavonoids were classified by the comparison with the data shown in Ferreres et al. 2008. SAP was further confirmed using analytical standard (98%, Extrasynthése, FR); * indicates fragments which were not observed in MS^2 by Ferreres et al. 2008 after fragmentation of parrent ions

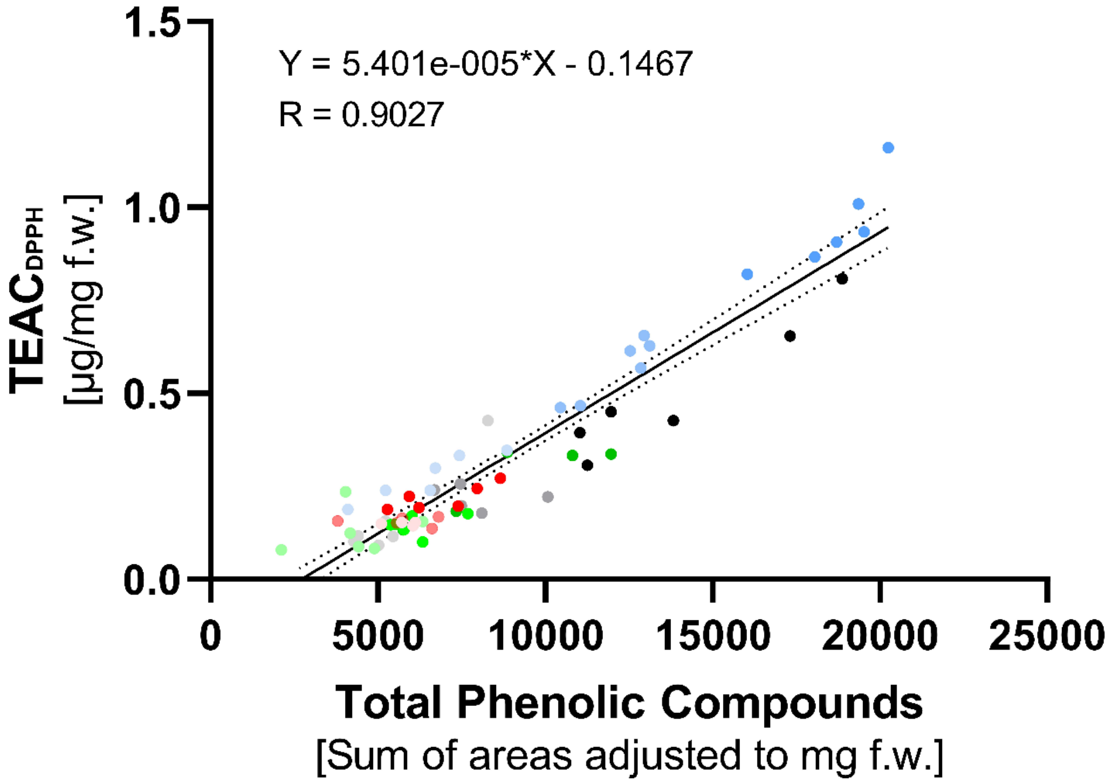

Figure S1: Correlation between total soluble PheCs content in leaf extract and its TEAC (Trolox Equivalent Antioxidant Capacity). Gray dots (white light), blue dots (blue light), red dots (red light), green dots (green light), light shade (low intensity, 100 µmol m^{-2} s^{-1}), medium shade (medium intensity, 200 µmol m^{-2} s^{-1}), dark shade (high intensity, 400 µmol m^{-2} s^{-1}), n = 5-6 ± SD.

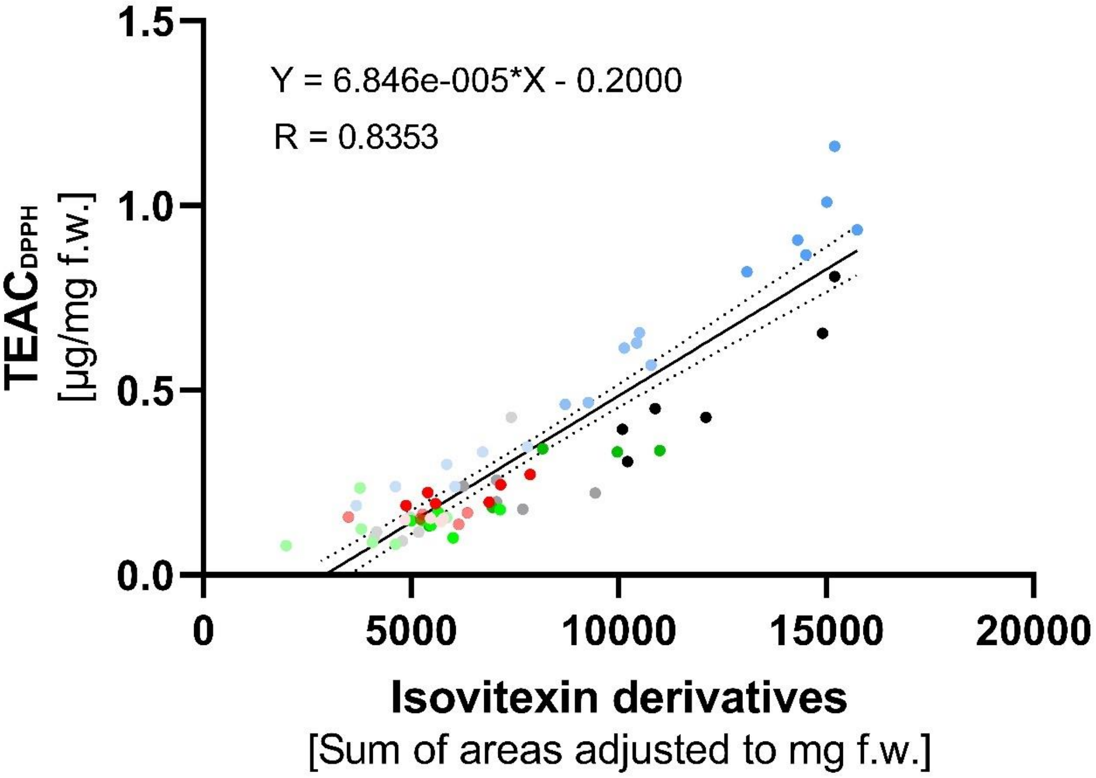

Figure S2: Correlation between content of isovitexin derivatives detected in leaf extracts and TEAC (Trolox Equivalent Antioxidant Capacity). Gray dots (white light), blue dots (blue light), red dots (red light), green dots (green light), light shade (low intensity, 100 µmol m^{-2} s^{-1}), medium shade (medium intensity, 200 µmol m^{-2} s^{-1}), dark shade (high intensity, 400 µmol m^{-2} s^{-1}), n = 5-6 ± SD.

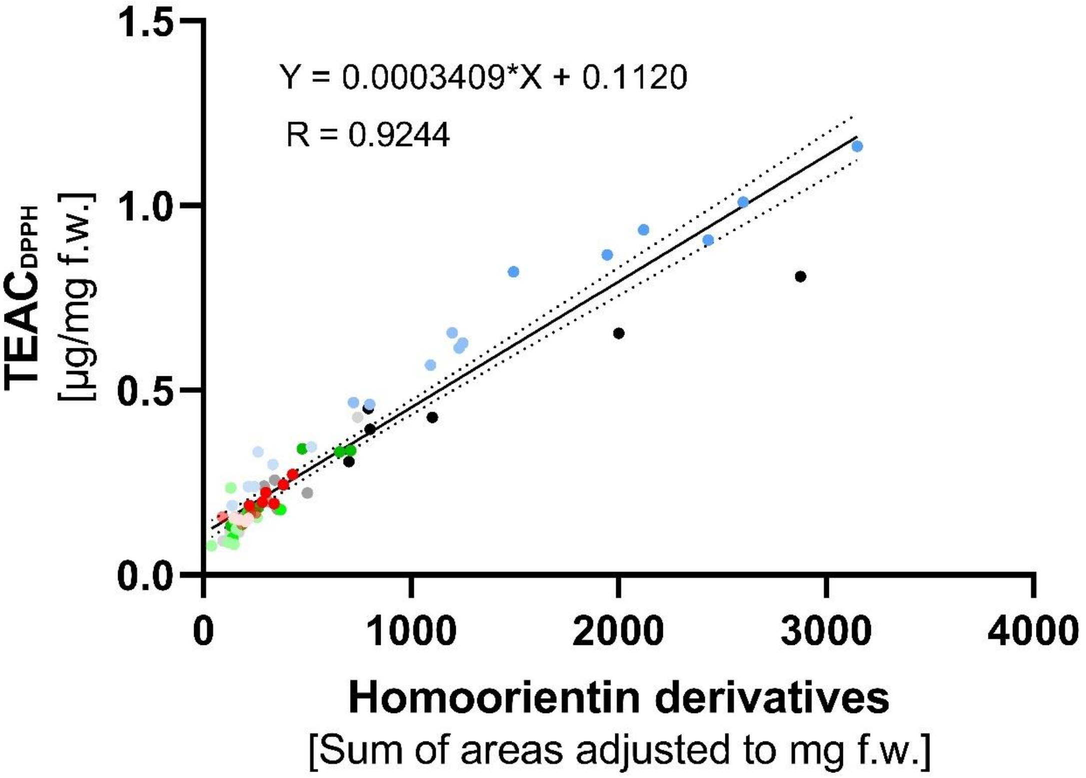

Figure S3: Correlation between content of homoorientin derivatives detected in leaf extracts and TEAC (Trolox Equivalent Antioxidant Capacity**).** Gray dots (white light), blue dots (blue light), red dots (red light), green dots (green light), light shade (low intensity, 100 µmol m^{-2} s^{-1}), medium shade (medium intensity, 200 µmol m^{-2} s^{-1}), dark shade (high intensity, 400 µmol m^{-2} s^{-1}), n = 5-6 ± SD.

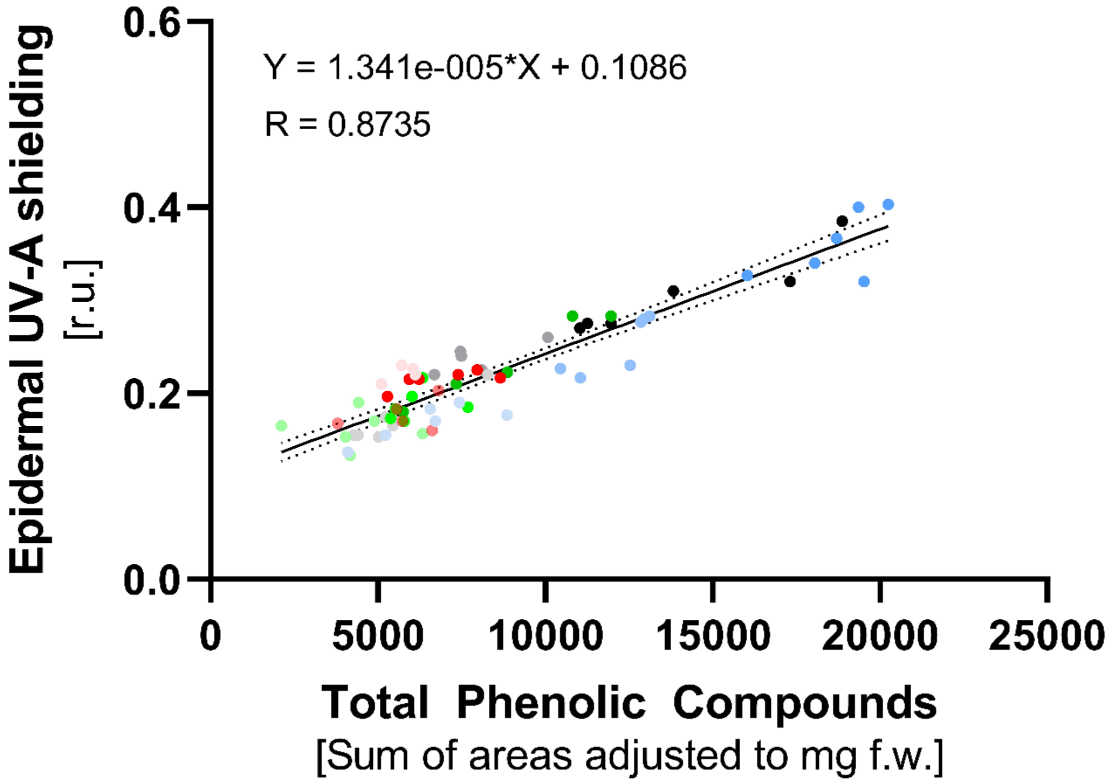

Figure S4: Correlation between content of total soluble PheCs detected in leaf extract and epidermal UV-A shielding. Gray dots (white light), blue dots (blue light), red dots (red light), green dots (green light), light shade (low intensity, 100 µmol m^{-2} s^{-1}), medium shade (medium intensity, 200 µmol m^{-2} s^{-1}), dark shade (high intensity, 400 µmol m^{-2} s^{-1}), n = 5-6 ± SD.

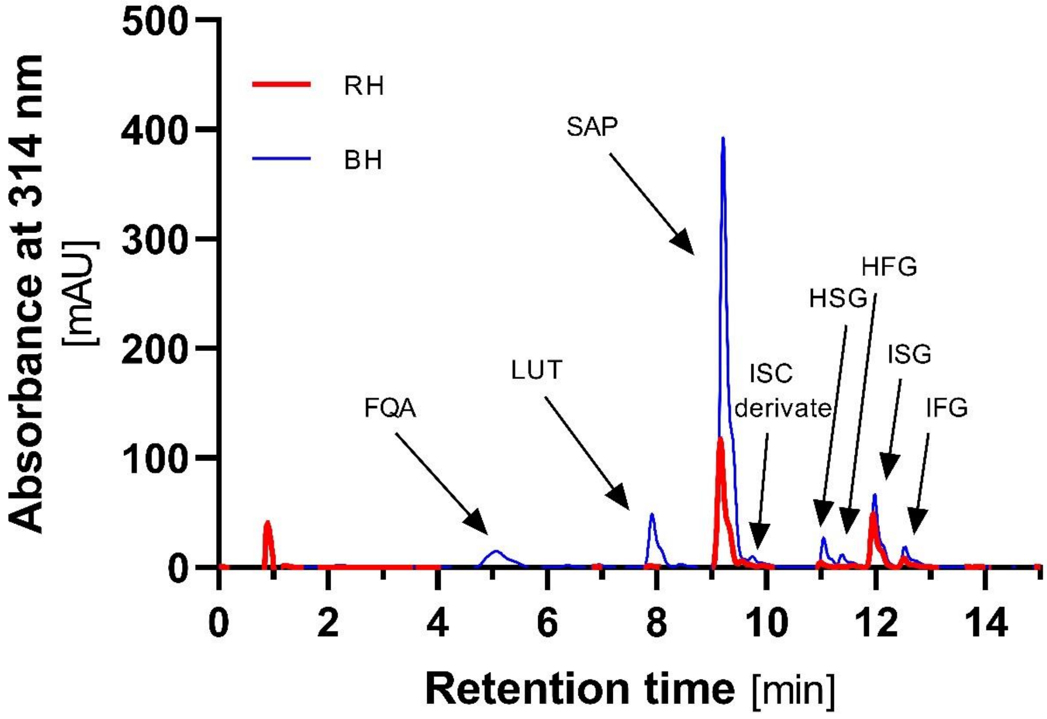

Figure S5: Typical chromatograms of separated phenolic compounds as obtain from HPLC-DAD analysis. Blue line (high intensity blue light treatment), red line (high intensity red light treatment). FQA (feruloylquinic acid), LUT (lutonarin), SAP (saponarin), ISC (isoscoparin derivative), HSG (homoorientin-7-O-[6-sinp]-glc), HFG (homoorientin-7-O-[6-fer]-glc), ISG (isovitexin-7-O-[6-sinp]-glc), IFG (isovitexin-7-O-[6-fer]-glc).

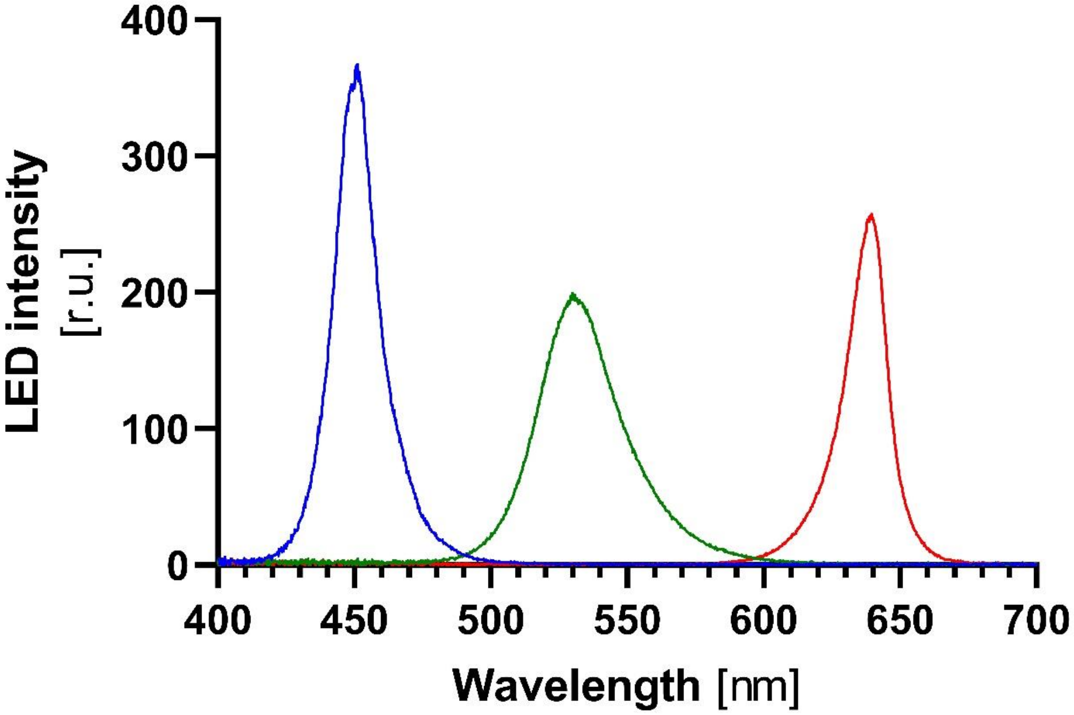

Figure S6: LEDs emission spectra measured approximately at the height of spring barely secondary leaves in growth chamber using Ocean Optics Spectrometer HR 4000CG-UV-NIR (Ocean Optics. USA). Blue line (blue LED), green line (green LED), red line (red LED).

References

[1] K. Masike *et al.*, "Highlighting mass spectrometric fragmentation differences and similarities between hydroxycinnamoyl-quinic acids and hydroxycinnamoyl-isocitric acids," *Chem. Cent. J.*, vol. 11, no. 1, p. 29, Dec. 2017, doi: 10.1186/s13065-017-0262-8.

[2] N. Kuhnert, R. Jaiswal, M. F. Matei, T. Sovdat, and S. Deshpande, "How to distinguish between feruloyl quinic acids and isoferuloyl quinic acids by liquid chromatography/tandem mass spectrometry: Distinguishing between feruloyl quinic and isoferuloyl quinic acids," *Rapid Commun. Mass Spectrom.*, vol. 24, no. 11, pp. 1575–1582, Jun. 2010, doi: 10.1002/rcm.4537.

[3] F. Ferreres, P. B. Andrade, P. Valentão, and A. Gil-Izquierdo, "Further knowledge on barley (Hordeum vulgare L.) leaves O-glycosyl-C-glycosyl flavones by liquid chromatography-UV diode-array detection-electrospray ionisation mass spectrometry," *J. Chromatogr. A*, vol. 1182, no. 1, pp. 56–64, Feb. 2008, doi: 10.1016/j.chroma.2007.12.070.

[4] S. Han *et al.*, "Systemic Responses of Barley to the 3-hydroxy-decanoyl-homoserine Lactone Producing Plant Beneficial Endophyte Acidovorax radicis N35," *Front. Plant Sci.*, vol. 7, 2016, Accessed: Apr. 25, 2022. [Online]. Available: https://www.frontiersin.org/article/10.3389/fpls.2016.01868

[5] A. Ghannam, H. Alek, S. Doumani, D. Mansour, and M. I. E. Arabi, "Deciphering the transcriptional regulation and spatiotemporal distribution of immunity response in barley to Pyrenophora graminea fungal invasion," *BMC Genomics*, vol. 17, no. 1, p. 256, Mar. 2016, doi: 10.1186/s12864-016-2573-x.

[6] O. Y. Shoeva, H.-P. Mock, T. V. Kukoeva, A. Börner, and E. K. Khlestkina, "Regulation of the Flavonoid Biosynthesis Pathway Genes in Purple and Black Grains of Hordeum vulgare," *PLOS ONE*, vol. 11, no. 10, p. e0163782, 10 2016, doi: 10.1371/journal.pone.0163782.

[7] J. Cai *et al.*, "Selection of appropriate reference genes for the detection of rhythmic gene expression via quantitative real-time PCR in Tibetan hulless barley," *PLOS ONE*, vol. 13, no. 1, p. e0190559, Jan. 2018, doi: 10.1371/journal.pone.0190559.

[8] E. SHAGIMARDANOVA *et al.*, "Oxidative Stress and Antioxidant Capacity in Barley Grown under Space Environment," *Biosci. Biotechnol. Biochem.*, vol. 74, no. 7, pp. 1479–1482, Jul. 2010, doi: 10.1271/bbb.100139.

[9] D. L. Parrott, J. M. Martin, and A. M. Fischer, "Analysis of barley (Hordeum vulgare) leaf senescence and protease gene expression: a family C1A cysteine protease is specifically induced under conditions characterized by high carbohydrate, but low to moderate nitrogen levels," *New Phytol.*, vol. 187, no. 2, pp. 313–331, 2010, doi: 10.1111/j.1469-8137.2010.03278.x.
